# Model-based reinforcement learning for ultrasound-driven autonomous microrobots

**DOI:** 10.1038/s42256-025-01054-2

**Published:** 2025-06-26

**Authors:** Mahmoud Medany, Lorenzo Piglia, Liam Achenbach, S. Karthik Mukkavilli, Daniel Ahmed

**Affiliations:** 1 Acoustic Robotics Systems Lab, Institute of Robotics and Intelligent Systems, Department of Mechanical and Process Engineering, ETH Zurich, Rüschlikon, Switzerland; 2 IBM Research - Europe, AI and Accelerated Discovery, Rüschlikon, Switzerland

**Keywords:** Mechanical engineering, Biomedical engineering

## Abstract

Reinforcement learning is emerging as a powerful tool for microrobots control, as it enables autonomous navigation in environments where classical control approaches fall short. However, applying reinforcement learning to microrobotics is difficult due to the need for large training datasets, the slow convergence in physical systems and poor generalizability across environments. These challenges are amplified in ultrasound-actuated microrobots, which require rapid, precise adjustments in high-dimensional action space, which are often too complex for human operators. Addressing these challenges requires sample-efficient algorithms that adapt from limited data while managing complex physical interactions. To meet these challenges, we implemented model-based reinforcement learning for autonomous control of an ultrasound-driven microrobot, which learns from recurrent imagined environments. Our non-invasive, AI-controlled microrobot offers precise propulsion and efficiently learns from images in data-scarce environments. On transitioning from a pretrained simulation environment, we achieved sample-efficient collision avoidance and channel navigation, reaching a 90% success rate in target navigation across various channels within an hour of fine-tuning. Moreover, our model initially generalized successfully in 50% of tasks in new environments, improving to over 90% with 30 min of further training. We further demonstrated real-time manipulation of microrobots in complex vasculatures under both static and flow conditions, thus underscoring the potential of AI to revolutionize microrobotics in biomedical applications.

## Abstract (web summary)

Medany et al. present AI-driven microrobots that use ultrasound propulsion to learn how to navigate complex environments. These microrobots achieve 90% success after minimal training and adapt rapidly, showing promise for biomedical applications.

## Main

Artificial intelligence (AI) has substantially advanced capabilities across a variety of fields, including diagnostics^1,2^, fluid mechanics^3,4^, medical imaging^5–7^ and segmentation^8,9^. AI has also played a crucial role in the development of sophisticated drone technology^10^ and autonomous vehicles^11^, reshaping our approach to transportation and surveillance. The integration of AI into microrobotics presents a new frontier^12–14^ and introduces distinct challenges for control and functionality.

Microrobotic manipulation offers groundbreaking possibilities, from microassembly to surgical tasks by improving localized drug delivery systems through precise manipulation within complex vasculatures under physiological conditions^15,16^. However, these applications demand high-precision microrobot control, which is complicated by their small size and complex dynamics. The manipulation of microrobots, therefore, presents unique challenges surpassing those of traditional robotics. Although autonomous vehicles can navigate reliably using established technologies like light detection and ranging and global positioning systems^11^, replicating these sensory capabilities at a microscale is exceptionally challenging. Microrobots often rely on imaging modalities, as most sensors are difficult to scale down or integrate. Furthermore, unlike autonomous vehicles powered by simpler motor controls, microrobots rely on less precise, wirelessly actuated systems such as those using light^17–19^, electricity^20^, chemistry^21–23^, magnetism^24–32^ or ultrasound^33–39^, complicating control further due to the influence of external forces.

The deployment of AI in microrobotics also confronts issues like overfitting, vulnerability to errors in new scenarios and long, often impractical training periods for model optimization. Reinforcement learning (RL)^40^ has proven to be a powerful tool and can enable robots to learn and adapt directly from their environment. Although RL has the potential to surpass human capabilities in tasks like object manipulation and strategic gameplay^41,42^, its reliance on extensive interactions for stable training introduces unique challenges for microrobots, as the experimental conditions are highly uncontrolled and variable.

Recent studies have explored a variety of AI-driven microrobots. Researchers have engineered light-driven microswimmers using Q-learning to navigate noisy environments and overcome challenges from Brownian motion^43^. Advances include the use of deep learning to autonomously steer magnetic swarms, which allows these microrobots to adjust their trajectories through channels of various dimensions by modifying their shapes^44^. Further innovations have seen the manipulation of magnetic microrobots in three dimensions using proximal policy optimization (PPO) following training in simulation environments^45^. Spiral magnetic microrobots have also been controlled using deep RL^46^.

Ultrasound-driven microrobots^47–51^, which have emerged as an exciting non-invasive alternative, are capable of generating tunable propulsive forces, enabling deep navigation into tissues. Nevertheless, achieving precise control and manipulation of these microrobots continues to pose substantial challenges, as several piezo-transducers (PZTs) need to be controlled with millisecond resolution for effective steering, a task often too complex for human operators. Recently, ultrasound microrobots have used Q-learning to navigate in a free environment^52^. However, such approaches lack generalizability, struggle in complex environments and do not account for flow or shapeshifting capabilities. Although adaptive methods have been used to control individual particles^53,54^, the training time required for manipulation using these algorithms increases exponentially with complexity. Despite this progress, the capabilities for autonomous obstacle avoidance and counter-flow navigation remain largely underexplored. Given the incomplete understanding of ultrasound microrobot behaviour, model-based reinforcement learning (MBRL) is a promising strategy for navigating them through complex environments with high precision. To date, no demonstrations of MBRL with ultrasound microrobots have been conducted.

**Fig. 1:**
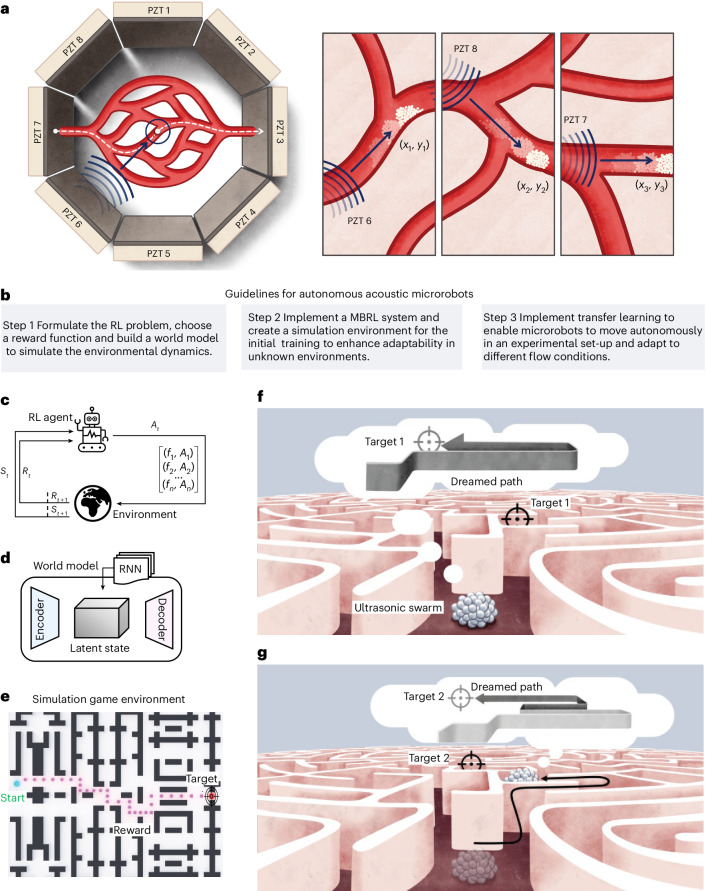
An autonomous ultrasound-driven microrobot. **a**, Left, schematic of the experimental set-up, which has an artificial vascular channel with eight PZTs in an octagonal configuration. Right, the schematic illustrates the behaviour of the microbot under ultrasound activation and details methods for its manipulation. **b**, Guidelines for manipulating microrobots. **c**, High-level formulation of the RL problem. The environment is our artificial channel. The RL agent is the microrobot with *S*_*t*_ actuated by the ultrasound frequency and amplitude to achieve a reward *R*_*t*_ after optimal actions *A*_*t*_. **d**, A world model encoder–decoder structure built to simulate and imagine future states in the environment. **e**, A simulated game environment designed to pretrain the microrobots, thereby reducing the convergence time during experimental training. **f**, A recurrent ‘dream’ in the latent space. The microrobot envisions several potential paths towards a target in a dreamed environment, allowing it to dream and train on various possible scenarios simultaneously. **g**, The microrobot agent executes the optimal action, successfully reaches target 1 and proceeds towards target 2 using a newly imagined path. *A*_*t*_, action, *R*_*t*_, reward; RNN, recurrent neural network; *S*_*t*_, state; ƒ_𝑛_, *A*_𝑛_, frequency and amplitude, respectively, of the 𝑛th US wave.

In this study, we employed the Dreamer v.3 MBRL^55^ algorithm to autonomously control an ultrasound-driven microrobot. Our approach integrates an in-house Python code for imaging and dynamic frequency adjustment of PZTs in an artificial vascular channel set-up (Fig. 1a). The code interfaces with an electronic circuit designed to allow rapid switching between transducers, a feature critical for navigational steering. Advanced image-processing techniques, such as the segment anything model^56^, are used to segment images, detect swarms and track them in real time. This approach frames control of the microrobots as an RL task that enhances their performance over time (Fig. 1b,c). To minimize the need for extensive physical experimentation, we implemented Dreamer v.3 to train within an imagined model (Fig. 1d) with an actor–critic RL architecture^57^. Although model convergence remains a challenge by taking up to 10 days, we developed a Pygame-based simulation environment (Fig. 1e) to accelerate the learning of essential navigation skills, such as path-planning and obstacle avoidance. This knowledge was then applied to enhance the adaptability of the system in physical experimental settings. The system was able to adapt in approximately 2 h. Within the simulation environment, we evaluated the performance of MBRL against state-of-the-art model-free RL algorithms^58^, which demonstrated the excellence of MBRL in managing complex channel navigations where model-free RL falls short. As previously discussed in research^55^, the ability of MBRL to imagine and simulate future actions, as shown in Fig. 1f,g, reduces the training time exponentially.

To address potential overfitting due to training on a single channel, we developed a general model trained across diverse channel environments, including vascular structures, racetracks and mazes. This model consistently delivered 90% accuracy across all trained channels. When tested on a new, previously unseen channel, the model initially achieved a 50% success rate, which notably increased to 90% after only 30 min of extra training. We conducted steering tests with microrobots in a stationary flow through several channels containing obstacles to thoroughly assess and demonstrate the effectiveness of the model. To optimize the model for dynamic flow conditions, we modified the reward function to enable the microrobots to adhere to walls and to navigate against the flow. These results highlight the potential of MBRL in advancing microrobotics for biomedical applications.

## Results

To investigate the control of microrobots through MBRL, we designed an experimental set-up that includes an artificial vascular channel encircled by eight PZTs set in an octagonal layout. To precisely control the activation and deactivation of the eight PZTs, we engineered a custom-built electronic circuit, achieving millisecond switching and integrated with a function generator. The artificial vascular channels were fabricated from transparent polydimethylsiloxane (PDMS) using standard mould replication and soft lithography^59^. The entire assembly was mounted on an inverted microscope. The experimental results—images and videos—were captured using a digital single-lens reflex camera recording between 6 and 18 frames per second. Image acquisition and processing were handled by in-house Python code.

Our microrobots were produced through the self-organization of commercially available, biocompatible microbubbles, each 2–5 µm in diameter, in an ultrasound field. These microbubbles were introduced into the channel by a liquid pump. In their quiescent state without ultrasound stimulation, the microbubbles remain randomly dispersed within the water solution. However, when subjected to an acoustic field, the microbubbles begin to scatter the sound waves. This scattering, coupled with the synchronized phase oscillation of adjacent microbubbles, triggers their self-assembly. For more details about the experimental set-up, refer to Supplementary Note 1.

To elucidate the steering mechanism of our microrobots positioned near PZT_6_ (Fig. 1a), we began by activating PZT_6_. This generated a pressure gradient between the microrobot and the transducer, which drove the microrobot from the higher-pressure area towards the lower-pressure region along the wave propagation path and perpendicular to transducer 6. Our experimental results show that the microbubbles were highly responsive to ultrasound; even at an excitation voltage of 4 V_pp_, velocities in the range of millimetres per second were achieved in a stationary flow. With the arrival of the microrobot at the designated checkpoint (*x*_1_, *y*_1_), we activated a second transducer, PZT_8_, while simultaneously deactivating PZT_6_. This action redirected the microrobot to align normal to PZT_8_ and guided it along the intended trajectory towards (*x*_2_, *y*_2_). We then activated a third transducer, PZT_7_, which steered the microrobot along a trajectory towards (*x*_3_, *y*_3_) while concurrently deactivating PZT_8_. This sequence of precise activations and deactivations of the PZTs enabled sophisticated control over the trajectory of the microrobot and facilitated complex navigational manoeuvres.

Achieving precise navigational control over the ultrasound-driven microrobot presents substantial challenges to a human operator, primarily due to the need for fast (milliseconds) and precise adjustment of the amplitude and frequency of the ultrasound signal, as well as the activation of various PZT elements. These adjustments influence the behaviour of the microrobot, which is often unpredictable. For example, proximity to a specific transducer necessitates a lower voltage to initiate motion, whereas a microrobot that is farther away requires a higher voltage to be mobilized. Similarly, smaller microrobots require less power, and larger ones need more. Furthermore, although the velocity of microrobots tends to scale linearly with voltage amplitude^52^, their response to frequency adjustments exhibits complex characteristics, including Gaussian distributions and several peaks, which indicates a varied response across different operational frequencies. Adding to the complexity, individual PZTs exhibit different frequency outputs, and the operational frequency range differs for each microrobot. This intricate interplay of control parameters underscores the complexity of managing microrobot movements within this advanced experimental framework. The variability in voltage, frequency and switching between PZTs introduces a substantial action space, which complicates the control process and necessitates an extensive amount of experimental data to effectively navigate this expanded action space.

**Fig. 2:**
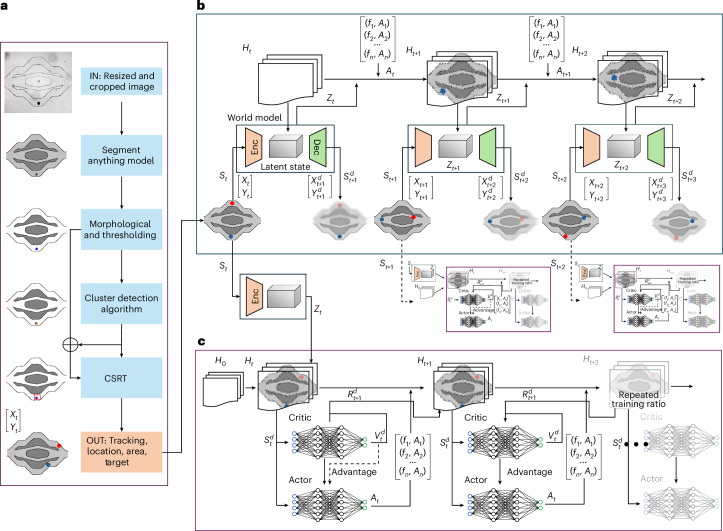
Model-based RL structure for an autonomous microrobot. **a**, The imaging pipeline processes channel images to track microrobot positions. It begins by resizing and cropping the images, followed by segmentation using the segment anything model. Morphological operations and thresholding refine the results. A cluster detection algorithm then identifies relevant clusters, and the CSRT monitors the position of the microbot, providing data on location, area and target information to the world model. **b**, The world model learns to encode the visual output from the imaging pipeline into a latent representation. It uses an encoder–decoder architecture to reconstruct the images, effectively capturing the latent state (*Z*_*t*_) from the input state (*S*_*t*_). The world model simulates future states (*S*_*t*+1_, *S*_*t*+2_, …) and actions (*A*_*t*_, *A*_*t*+1_, …) in the latent space, facilitating the prediction of microrobot movements. **c**, The actor–critic network is iteratively trained within this loop to optimize the policy. It operates in the ‘dreamed’ environment simulated by the world model. This approach reduces the reliance on real-time interactions with the experimental environment. Dec, decoder; Enc, encoder.

This vast action space necessitates the implementation of an MBRL strategy. Our approach begins by feeding the MBRL model an image of the vascular channel following any PZT activation. This image acts as feedback for our MBRL model, enabling it to assess the current state of the microrobot within the experiment. We then apply advanced image-processing techniques to detect and track the movements of the microbot, as shown in Fig. 2a and Methods.

### Model-based RL in microrobotics

In our experimental set-up, we employed MBRL to address challenges associated with microrobot manipulation in a complex microvasculature environment. The control problem for the microrobot was formulated using an RL framework in which the state space is defined by image data and the action space as either discrete or continuous variations in frequency, amplitude and the number of PZT activations. Each action involves adjustments to the amplitude and frequency of a single, specific PZT at a time. Rewards are calibrated based on the efficacy of the microbot in reaching a target. Details are provided in Methods.

To augment the MBRL set-up, we chose the Dreamer v.3 algorithm^55^, which is known for its adept handling of high-dimensional state spaces and complex dynamical systems. This algorithm integrates three primary components: world model learning, envisioning possible future scenarios (Fig. 2b) and applying RL to these scenarios (Fig. 2c). Together, these elements construct a latent (hidden) model of the environment that predicts or simulates future trajectories, which assists in training decision-making networks with these predictions (Methods). This approach is particularly valuable as it reduces the reliance on extensive physical data, which is beneficial in scenarios like microscale operations where data acquisition is challenging. This MBRL set-up not only enhances the precision of microrobot control but also optimizes learning efficiency, making it a quintessential tool for advancing robotic interventions in microvascular environments.

### Simulation environment

We used MBRL to train our model using experimental data over a period of 6 h. Despite this effort, the performance of the model did not improve, which we attributed to uncertainties surrounding the optimal reward function for this set-up. To reduce the need for repeated physical experiments while testing various reward functions, we developed a Pygame^60^-based simulation environment to model the behaviour of the microrobot. Pygame is a versatile library used to create interactive game environments, which we used to simulate dynamic and interactive environments for our microrobots. This environment focuses primarily on local path-planning and obstacle avoidance, intentionally omitting the complex dynamics of microrobots, such as PZT resonance and microbubble size, which are intended for exploration in future experimental set-ups.

**Fig. 3:**
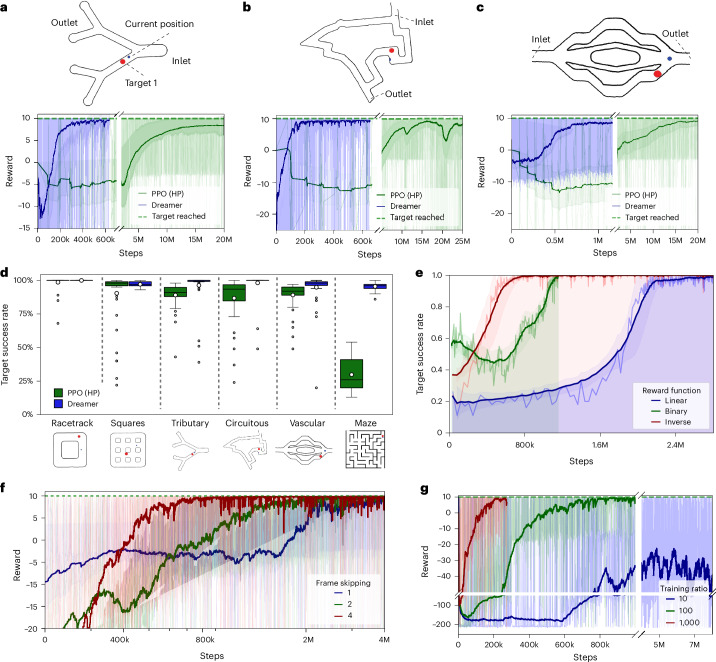
Performance analysis of microrobot navigation in various environments using RL algorithms. **a**–**c**, Reward trajectories for a multi-output tributary channel (**a**), a circuitous channel (**b**) and a vascular channel (**c**). Dreamer v.3 (blue) outperformed the hyperparameter-tuned state-of-the-art PPO (green) across simulation steps. Solid lines show smoothed rewards using an exponentially weighted moving average (EWMA; *α* = 0.002), and shaded areas represent ±1/3 of the standard deviation around the smoothed data. **d**, Comparison of the PPO and Dreamer algorithms in reaching targets in different channel types: racetrack, tributary (four-out), circuitous, squares, vascular and maze. Box plots show the rate of target achievement evaluated over the final 50 episodes for each trained policy. Boxes represent the interquartile range (IQR; 25th–75th percentiles), the central line indicates the median, whiskers extend to 1.5 × IQR and individual points show outliers. White circles denote the mean. **e**, Impact of different reward functions on the rate of target achievement. Solid lines represent the centred EWMA of the rate of target achievement (*α* = 0.1). Shaded regions denote ±1 standard deviation around the EWMA. **f**, Effects of frame-skipping on performance, presented as a logarithmic plot. Solid lines represent the EWMA of the reward (*α* = 0.0015), with shaded regions showing ±1/3 of a standard deviation. **g**, Influence of training ratio on reward dynamics, highlighting consistent performance across various ratios in simulated environments. Solid lines represent the EWMA of the reward (*α* = 0.008), with shaded regions indicating ±1/3 of a standard deviation. HP, hyperparameter.

The simulation environment is rendered as a 64 × 64 RGB image, structured within a ‘gymnasium.spaces.Box’^61^ with dimensions of (64, 64, 3). In this image, obstacles are distinctly marked in dark grey, channels in white, target points in red and the agent’s position in blue. The agent is depicted with a circular red dot, designed to mimic the microbubble clusters observed in physical experimental scenarios. We evaluated the performance of MBRL (Dreamer v.3) against PPO^58^, a state-of-the-art model-free RL algorithm. Our findings highlight that MBRL exhibited superior efficiency and adaptability within our specific environment, as shown in Fig. 3.

Figure 3a demonstrates that in a simple multi-output tributary channel, both algorithms reached convergence; however, our model converged ~50 times faster than the hyperparameter-tuned PPO. Figure 3b shows that for a circuitous racetrack, PPO required approximately 25 million steps to converge, whereas MBRL achieved convergence in just 600,000 steps. Similarly, in the vascular channel shown in Fig. 3c, MBRL converged after 1 million steps, whereas PPO required around 25 million steps. Overall, our MBRL approach consistently demonstrated faster convergence than the hyperparameter-tuned PPO across all tested environments, including a complex maze (Fig. 3d). We experimented with various reward functions, including binary, inverse and logarithmic (Supplementary Note 2). Figure 3e compares these reward functions and highlights that the motivation for using an inverse reward function is to achieve the fastest convergence. This simulated setting enabled us to refine and iterate on our reward functions and control strategies efficiently, without the continuous need for live experimental adjustments (Methods).

### Implementation of action space for MBRL

To effectively train the microrobots, we began by assessing potential combinations of actuator frequencies and amplitudes through experiments, which revealed a large action space with four frequency inputs, four amplitude inputs and four PZT activation units, resulting in 64 distinct combinations. After a thorough analysis of the experimental data, we developed an amplitude predictor designed to tailor the ultrasound field intensity to the size of the microrobot. This allowed us to refine our approach by eliminating four amplitude options, thereby reducing our action space to 16 precise settings (Supplementary Note 2). The optimization led to precise control over the microrobot velocity and overshooting based on the size of the microrobot. We further optimized performance by selecting operational frequencies between 2.7 and 2.9 MHz, which align closely with the resonant frequencies of the PZT.

To enhance the efficiency of our MBRL model, we implemented frame-skipping^41,62^ to reduce the computational load and accelerate training without compromising performance. This method notably reduces the number of frames processed by the MBRL agent, allowing the model to focus on noticeable changes and minimize the risk of overfitting. Additionally, we implemented max pooling (selecting the maximum pixel value across the skipped frames) across the last two frames to decrease the temporal resolution while maintaining essential dependencies between skipped frames. This adjustment greatly enhanced training stability, resulting in smoother convergence, faster learning and improved overall task performance. For our experiments, after testing different frame-skipping rates, we opted to skip four frames to achieve faster convergence (Fig. 3f). Although higher frame-skipping rates lead to quicker convergence, they also result in overshooting, as detailed in Supplementary Note 9. We also explored a critical parameter known as the ‘training ratio’, which is the number of steps trained in the ‘imagination’ of the world model versus experimental environment steps. Using higher training ratios (1,000:1) reduced the need for experimental interactions and enhanced learning efficiency by allowing the agent to learn from less costly imagined experiences. Although a lower ratio (1:1) provided more precise feedback, it slowed learning (Fig. 3g and Methods). To maximize the benefits of high training ratios, we developed a parallel script to run experimental environment interactions in the simulation or in the physical set-up and the world model training on separate threads. We implemented an adaptive training ratio that was dynamically adjusted based on the agent’s performance in the real environment.

### Transition of autonomous microrobots from simulation to physical environment

**Fig. 4:**
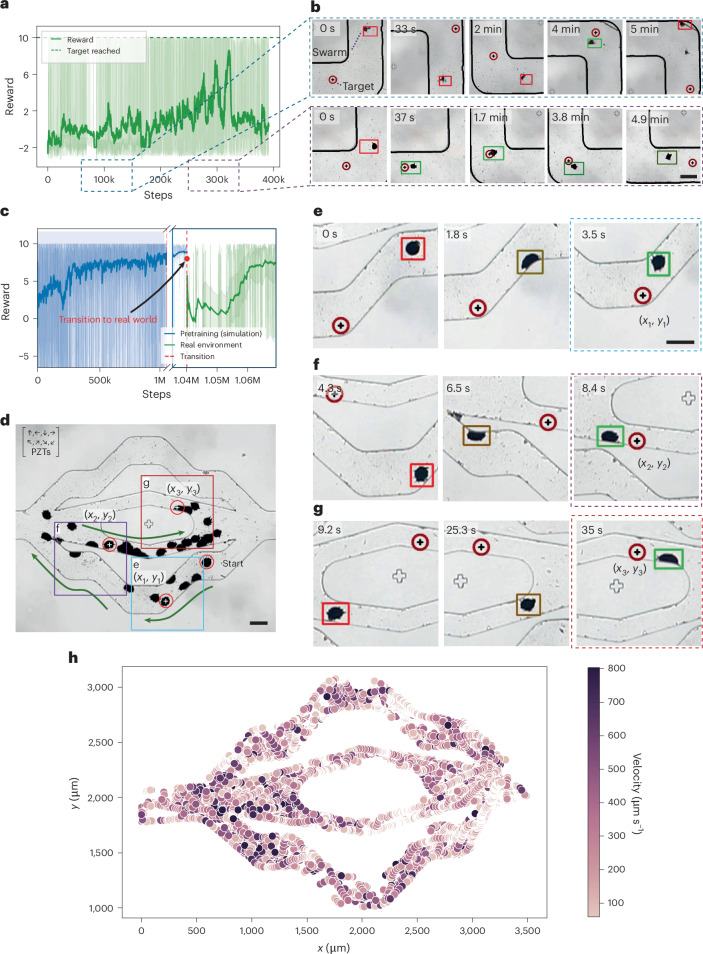
Transition of autonomous microrobots from the simulation environment to the physical environment. **a**, MBRL plot showing the training of microrobots on 32 actions in an experimental racetrack with a square channel. The *y* axis indicates the reward function, with positive values signalling successful navigation towards the target and negative values indicating crashes. The *x* axis displays the number of training steps. The solid line shows the EWMA (*α* = 0.01), and the shaded area represents ±0.5 of the rolling standard deviation (window size of 50) around the EWMA. The reward drops notably at around 300,000 steps due to overfitting to a specific corner of the channel. **b**, Top, image sequence illustrating the experimental training of microrobots after 100,000 steps. At this early stage, the microrobots frequently failed and reached the target only 20% of the time. Bottom, image sequence showing that after 350,000 training steps, the microrobots demonstrate improved efficiency, reaching the target faster (in seconds) 75% of the time. Red boxes mark the microrobot when it is far from the target, whereas green boxes indicate proximity to the target. The target position is denoted by a circle with a black plus symbol. **c**, Plot of RL performance illustrating the transition of a pretrained MBRL model from the simulation environment to the physical environment. The score rapidly increased during the simulation phase (blue), dropped after the transition to experiments (marked by a red vertical dashed line) and then increased as the model adapted to the physical environment (green). Solid lines represent the EWMA (*α* = 0.01) of the reward, with shaded regions indicating ±0.5 of the rolling standard deviation (window size of 50) around the EWMA. **d**, Micrograph with superimposed images of the navigation steps of microbots within an artificial vasculature. The start and target points are marked by red hollow circles. Arrows in the top left corner indicate all possible movement directions of the microrobots. **e**–**g**, Image sequences demonstrating a microrobot navigating an artificial vasculature, starting from the initial position and sequentially reaching three predefined targets: (*x*_1_, *y*_1_) (**e**), (*x*_2_, *y*_2_) (**f**) and (*x*_3_, *y*_3_) (**g**). **h**, Heat map depicting the relation between speed and position within an artificial vasculature channel. The colour gradient indicates the speed at each position, with darker colours indicating higher speeds and lighter shades denoting slower velocities. Scale bars, 200 µm.

Pretrained models in a simulation environment have been highly effective in our physical experiments, adeptly handling tasks like path-planning and localization. Our main task was to adjust these models to control the frequencies and amplitudes needed to direct the microrobots in the new settings. A model trained solely on experimental images was deployed in a racetrack channel, as shown in Fig. 4a,b. It achieved approximately 70% of our target objectives within 10 days of continuous operation, but it tended to overfit, causing the microrobot to remain within certain sections of the channel (Supplementary Video 1). Forcing the microrobots to navigate to different areas of the channel resulted in decreased performance and a failure to reach the designated targets. This behaviour underscores the need for further model adaptation to minimize overfitting. It also highlights the value of incorporating pretraining in a simulated environment, which can substantially reduce training time and improve the overall adaptability of the model (Supplementary Video 2).

### Mapping continuous actions within a discrete framework

Although pretraining on discrete actions initially offered benefits, it was constrained by the limited action space, as we could choose from only four frequencies, with various responses across PZTs. We realized the importance of determining the optimal frequency for each PZT to ensure effective microrobot navigation at each channel point. To overcome these challenges and facilitate precise adjustments in frequency and amplitude, we implemented a continuous action space. This space includes frequency values ranging from 2.7 to 2.9 MHz and amplitudes from 4 to 14 V_pp_, selections informed by manual control experiments. Additionally, we incorporated the rapidly exploring random tree star algorithm for path-planning, which ensured optimal navigation paths. By activating the PZT opposite to the desired direction of movement, we were able to focus exclusively on learning the nuances of continuous actions for frequency and amplitude.

**Extended Data Fig. 1:**
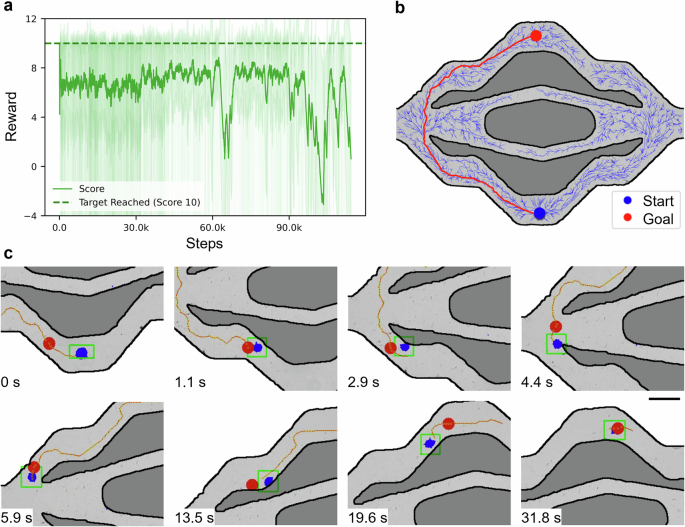
Training with Continuous Actions. **a**. A plot displaying the relationship between the reward and the steps, illustrating the training progress and performance trends. The solid line represents the exponentially weighted moving average (EWMA) of the reward (α = 0.01), and the shaded region shows ±½ of the rolling standard deviation (window = 50). **b**. RRT* path planning within an artificial vascular channel. **c**. A sequence of images showing the microrobot (blue) following the preplanned path (yellow) to reach updated targets (red). The updated targets and intermediate points are marked along the path to visualize the microrobot’s tracking accuracy and performance. Scale bar: 300 µm.

To ensure that the agent consistently followed the designated paths, we refined the reward function, triggering a reset and initiating a new episode whenever notable deviations from the designed path occurred. During training, we observed a saturation of the reward function, primarily due to repeated frequency adjustments. These adjustments often caused the microrobots to overshoot their targets, resulting in erratic movements. Furthermore, the model frequently opted for higher amplitudes in an attempt to accelerate target acquisition. Although this approach initially seemed advantageous, it typically increased navigation instability, as the microbot further deviated from the target point. The frequent fluctuations in both frequency and amplitude compromised precise control, making it challenging to get a microbot to accurately adhere to the designated paths. The complex and dynamic environment coupled with the nonlinearities of the system and the intricate action space presented major challenges in achieving consistent, stable performance with the model. Despite these challenges in integrating continuous action with path-planning, we managed to navigate the microrobots, although not perfectly (Extended Data Fig. 1 and Supplementary Video 3).

In response, we incorporated a sweeping action around the resonant frequency of the PZT into each discrete action using our programmable function generator set to steps of 1 ms. This strategy leveraged the resonant frequency characteristics of the PZTs to ensure that microrobots consistently operated at or near their optimal frequencies. By using pretrained simulation environments, we enhanced performance and reduced experimental time (Fig. 4c and Supplementary Video 4). The implementation of sweeping actions resulted in smoother transitions and more stable movements, thus addressing the overshooting observed during the continuous training phase, as shown in Fig. 4d–g. This refined approach represents a substantial advance in microrobot navigation as it enables more precise and effective control. Figure 4h presents a heat map illustrating the relation between speed and position within an artificial vasculature channel.

### Dynamic adaptation of MBRL to complex and variable environments

**Fig. 5:**
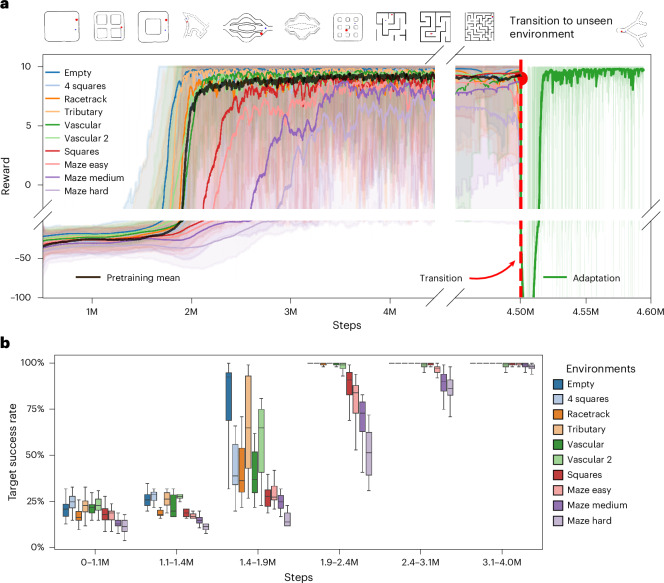
Performance of the generalized world model across various environments. **a**, Reward progression over number of training steps across ten distinct environments, including empty, four squares, racetrack, vascular and several maze configurations. Each coloured line represents the centred EWMA (*α* = 0.002) of the reward for each environment, with shaded regions indicating ±0.5 of the rolling standard deviation (window size of 1,000 steps). Convergence was faster in simpler environments, whereas more complex ones required extra training steps. After 4.5 million steps, marked by a vertical dashed red line, the model transitioned from pretraining across the ten simulation environments to adaptation within a new multi-output tributary channel. The black curve denotes the average pretraining performance across all environments. Following the transition, the model adapted rapidly, achieving stable performance within ~50,000 steps (approximately 30 min) in the new environment. **b**, Success rate of targets reached across different environments plotted against number of training steps. The box plots illustrate the variability and distribution of the performance of the MBRL algorithm in successfully reaching targets. Although simpler environments facilitated quicker convergence, our MBRL model consistently attained convergence across all scenarios. Steps are grouped into logarithmic bins from 0 to 4 million steps, and each box summarizes target-reaching rates across a training run within each bin. Boxes indicate the IQR, the horizontal line marks the median, whiskers extend to 1.5 × IQR and outliers are omitted for clarity.

**Extended Data Fig. 2:**
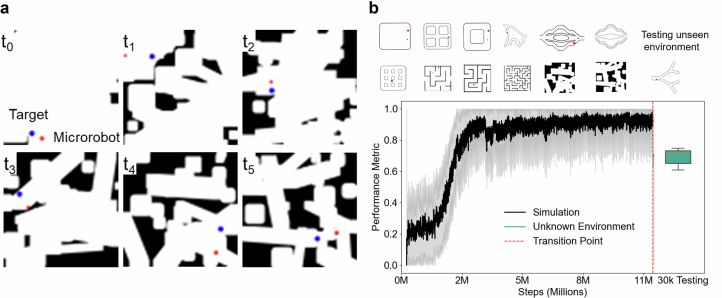
MBRL Model Training and Adaptation. **a**. Simulated Randomized Environment Complexity: Displays six images, each at a different time step, showing the increasing complexity of simulated randomized environments with obstacles (black) and paths (white). The microrobot (red) navigates towards the target (blue) through these evolving challenges. **b**. Generalization Across Environments: Depicts adaptation over 12 environments, including 10 original plus 2 newly introduced randomized environments, culminating in a 70% success rate in a novel testing environment, highlighting effective generalization. Solid lines show the exponentially weighted moving average (EWMA, α = 0.01) of success rates, with shaded areas indicating ±½ of the rolling standard deviation (window = 50 steps). A red dashed line marks the environment transition. The final box plot shows the post-adaptation performance, with boxes representing the 25th–75th percentile and whiskers extending to 1.5×IQR.

We have demonstrated that our MBRL model, once trained to navigate in a specific environment, can generalize to diverse environments through fine-tuning. After the transition from a tributary channel configuration (Supplementary Fig. 16) to an unseen vascular environment, ~400,000 training steps were required to achieve over a 90% success rate. To reduce the adaptation time frame and prevent overfitting to any single training scenario, we exposed the model to a variety of environments, including various vascular networks, mazes and racing circuits. Figure 5a illustrates the reward function and the target success score across ten mixed environments. Subsequently, the model was able to adapt to an entirely unknown environment, such as a multi-output tributary channel, within about 50,000 steps or roughly 30 min (Supplementary Video 5). Moreover, Fig. 5b quantitatively illustrates the ability of the model to achieve target objectives across a range of training environments for various numbers of training steps. The success rate improved from 25% to 100% as the model accrued more training across environments, ranging from simple constructs like empty and quadrant channel to more complex configurations such as maze medium and maze hard. Between 3.1 million and 4 million steps, it consistently reached and maintained a success rate above 90% across all environments, demonstrating its ability to effectively converge and adapt to diverse channel dynamics. This confirms that the world model accurately captured the dynamics and demonstrated robust and reliable performance. Furthermore, we introduced two randomized environments alongside the ten environments during training. We dynamically altered the layout of obstacles by converting white pixels to black, thereby generating unpredictable maps. As training progressed, the complexity of these randomized environments was gradually increased. More obstacles were introduced to intensify the training challenge. After 11 million training steps, the model achieved a 70% success rate in completely unseen environments, highlighting its enhanced adaptability (Extended Data Fig. 2). However, note that as the complexity of the environments was increased, the convergence time of the model tended to lengthen.

### Autonomous manipulation in a physiological flow

Autonomous navigation and manipulation within dynamic flow environments present substantial challenges for microrobotics^63–65^. Initially, our models were trained under no-flow conditions within a vascular channel; however, when these models were subsequently applied to flow conditions, they faced considerable difficulties due to the increased drag forces. The flow often flushed the microrobots away, necessitating substantial manual effort to restart the training and assembly of the microrobots. Furthermore, the pretrained model was now less effective due to the bigger domain gap between the simulation and the physical environment.

**Fig. 6:**
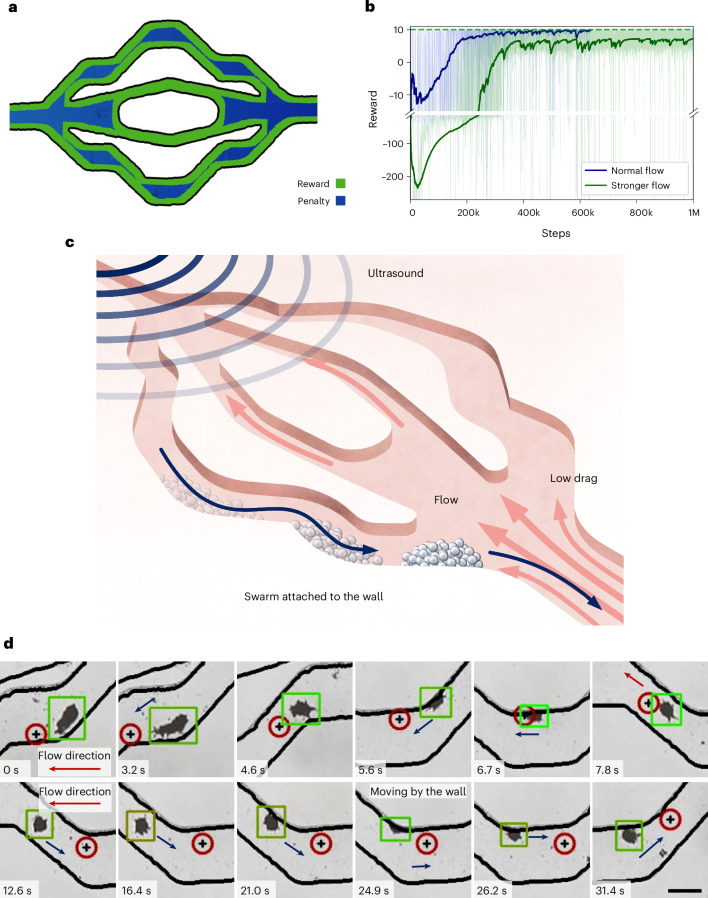
Autonomous navigation of a microrobot upstream in a flow environment. **a**, Schematic of the reward function adjusted to promote microrobot navigation close to the wall to minimize drag. **b**, Graph showing reward progression over time for microrobots in normal (blue line) and stronger (green line) flow conditions, highlighting differences in learning and adaptation. Solid lines represent the EWMAs (*α* = 0.0015) of the reward. In the normal flow, rewards steadily improve and stabilize around 200,000 steps. In the stronger flow, initial difficulties lead to more negative rewards, but the algorithm shows notable improvement by 400,000 steps. **c**, Schematic illustrating the behaviour of the microrobot attached to the wall to avoid drag and move against the flow. **d**, Image sequence showing a microrobot navigating within a microfluidic channel under flow conditions. Initially, when the microrobot was at the centre of the channel (0.0 to 6.7 s), it encountered maximum drag before moving towards the wall (7.8 to 31.4 s), where the drag was minimal. The reduced drag forces at the wall facilitated more stable and controlled navigation. The red arrows indicate the direction of the fluid flow. Scale bar, 200 µm.

First, we adjusted the reward function to impose penalties for microrobots moving into the centre of the channel (Methods and Supplementary Note 5), where drag forces are typically strongest (Fig. 6a). We also incorporated a simulated force in our environment that continuously pushes against the microrobots, quantified in pixel values corresponding to the flow rate we aimed to counteract. As shown in Fig. 6b, more steps are required to achieve convergence in a stronger flow. These adjustments produced a more realistic simulation of the physical challenges encountered by microrobots in flow conditions. Finally, we refined the physical model of microbubble dynamics, focusing particularly on bubble–wall interactions. Specifically, the secondary Bjerknes force attracts the microbubble cluster to the wall leading to subsequent adhesion to the channel walls (Fig. 6c). The cluster then benefits from the ‘no-slip’ condition at the wall, which reduces the shear forces and facilitates easier movement along the wall.

In addition to environmental characteristics, the response of microrobots to acoustic actuation—particularly how they react linearly to changes in voltage and cluster size—necessitated adjustments to the amplitude predictor. Our strategy for navigating environments with flow involved increasing the power when moving against the flow to counteract the increased drag and reducing the power when moving with the flow to take advantage of the reduced resistance. This differential power strategy led to more efficient navigation and manipulation within complex flow environments (Fig. 6d and Supplementary Video 6). Finally, for when we lose sight of the microrobots, we implemented a rescue function that accesses their last coordinates and attempts to reverse the recent actions to return the microrobots to our field of view (Supplementary Note 9).

## Discussion

Although ultrasound microrobots offer important advantages for biomedical applications, controlling them remains a bottleneck. In this study, we demonstrate steerability of microrobots in complex channels using only ultrasound. We could guide them autonomously against a flow in real time using state-of-the-art MBRL strategies. Moreover, we show that incorporating a simulation environment accelerated this process. After transitioning from a pretrained simulation environment, we achieved sample-efficient collision avoidance and channel navigation, reaching a 90% success rate in target navigation across various channels within an hour of fine-tuning. Additionally, our model initially generalized successfully in 50% of tasks in new unseen environments, improving to over 90% with 30 min of further training. Furthermore, to facilitate motion in a flow environment, we adjusted our simulation set-up based on fluid dynamics by exploiting low-drag regions near the wall and the attractive forces between the microrobots and the wall. This enabled real-time navigation both against and with the flow, underscoring the potential of AI to revolutionize microrobotics in biomedical applications.

**Extended Data Fig. 3:**
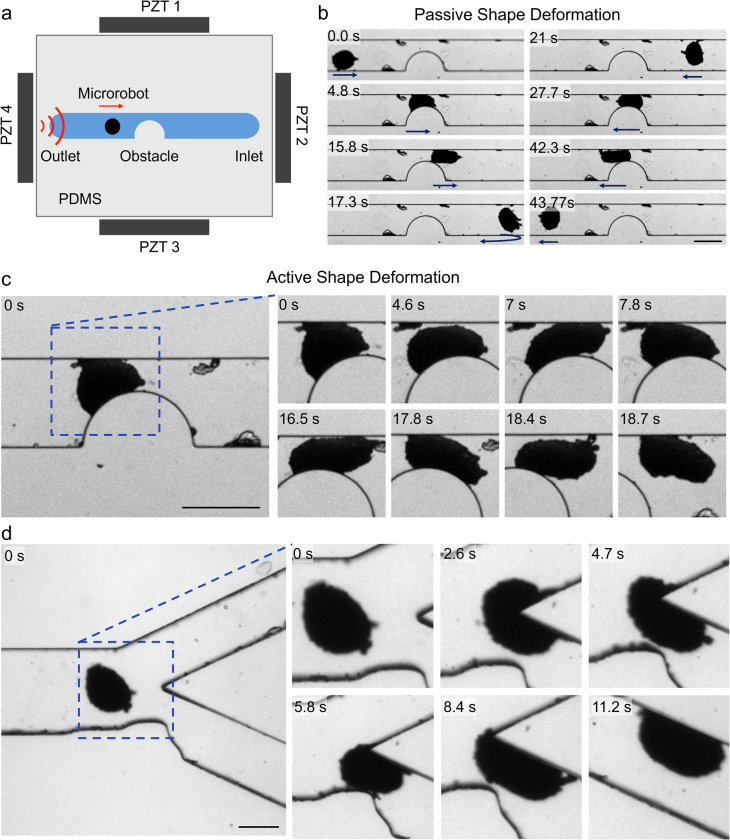
Microrobot Active and passive Shape-shifting, Demonstrated in Various Configurations. **a**. Schematic of the experimental setup, showing a straight microchannel equipped with four piezoelectric transducers (PZTs) positioned around an obstacle to manipulate the microrobot. **b**. Sequence of images illustrating passive microrobot deformation as it navigates through an obstacle using a single PZT activated along the X-axis at 20 volts, demonstrating the robot's ability to passively overcome the barrier. **c**. Series of images showing active manipulation where two PZTs on the Y-axis are activated, detailing the microrobot's dynamic shape deformation to navigate around the obstacle. **d**. Image sequence in a bifurcation channel setup where the microrobot encounters an obstacle on one side and actively shape-shifts to maneuver towards the downward path, showcasing advanced control and navigational capabilities. Scale bar: 100 µm.

We envision our work being applied across a range of manipulation strategies within microfluidics. It could enhance single-cell studies and facilitate research on small animal models such as *Caenorhabditis* *elegans*^33^ and zebrafish embryos^66^. Additionally, our techniques could substantially advance microparticle separation and other precision applications in biotechnology and healthcare. Beyond these uses, the framework could drive innovations in minimally invasive surgical procedures. This includes the use of ultrasound-driven microrobots, such as shape-morphing^47^ and spiral^48^ designs, as well as those propelled by streaming forces^51^, possibly leading to innovative solutions for medical interventions. Preliminary experiments with passive and active dynamic shapeshifting (Extended Data Fig. 3 and Supplementary Video 7) further demonstrate how microrobots can adapt to obstacles in real time, a critical capability for navigating living systems. We also explored how these microrobots adapt and navigate in bifurcated and vascular-like channels using ultrasound. Future efforts will extend our MBRL framework to shape control tasks by either training policies directly on experimental data or leveraging physics-based simulations to pretrain adaptive behaviours under complex acoustic interactions. For the microrobotics community, our image-based model, once initialized with specific actuator settings, can be easily adapted for actuation systems based on light^17–19^, chemistry^21–23^, electricity^20^ and magnetism^30–32^, thereby enabling autonomous control of diverse microscale objects across various experimental contexts.

**Extended Data Fig. 4:**
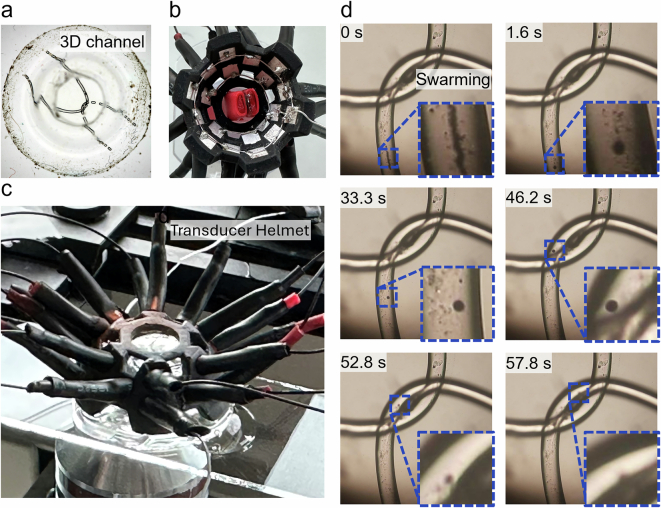
3D Demonstration of Microrobot Manipulation. **a**. 3D microchannel where microrobots are manipulated. **b**. Bottom view of the transducer array, illustrating the arrangement of PZTs for 3D control. **c**. Experimental setup featuring an array of 18 piezoelectric transducers placed in a conical channel, with coupling gel applied between the array and the microchannel for efficient ultrasound transmission. **d**. Sequence of images showing microrobot movement in 3D as it leaves the focal plane, demonstrating its ability to navigate beyond the primary field of view.

Looking ahead, we anticipate expanding our work into three-dimensional (3D) manipulation by integrating several cameras and optimizing the imaging pipeline for 3D data acquisition. Initial experiments have already successfully demonstrated 3D microrobot manipulation (Extended Data Fig. 4).

However, integrating several microscopes at different angles remains technically challenging due to the micrometric size of the microrobots. Addressing these challenges may allow the application of MBRL to 3D navigation. Building on these foundations, future work will focus on developing fully automated 3D control systems and advancing AI-driven shapeshifting capabilities that dynamically adapt to environmental stimuli. Integrating medical imaging techniques, such as ultrasound^67^ and two-photon microscopy, may allow us to study microrobot behaviour in animal models more effectively^14^. Achieving complex manoeuvring and task execution in 3D spaces will require a more sophisticated actuator system. With these technological advances, we aim to streamline the process for in vivo testing by using state-of-the-art segmentation methods in medical applications^8,9^, starting with animal models like mice^68,69^ and eventually scaling up to larger mammalian models. Future developments will also focus on reducing the dependency on expert oversight by creating user-friendly software interfaces and further improving the robustness of the model to facilitate its application in clinical settings.

## Methods

### Microchannel fabrication

The microfluidic channels used in the study were produced through standard soft lithography with PDMS. Each device was fabricated using a master mould and lithographically patterned with an SU-8 negative photoresist on a 4-inch silicon wafer, which was later placed inside a Petri dish. The thermocurable PDMS prepolymer was prepared by mixing the curing agent with the base at a weight ratio of 10:1. After degassing under vacuum, the prepolymer was cast onto the mould. PDMS was crosslinked by thermal curing for 2 h at 85 °C. The PDMS was poured into the mould and then cut and peeled from the channel mould. A 0.75-mm punch was used to punch the inlet and outlet ports. The ports were created by mounting the puncher at an angle of 60°, which prevented fluid from entering the channel at an angle that may cause a break in the plasma treatment bond between the layers of PDMS, which could result in leakage and the malfunctioning of the environment. Another PDMS layer was bonded onto the PDMS channel by plasma treatment for 1 min, followed by curing at 85 °C for 2 h. A PZT was attached to the PDMS channel wall orthogonal to the aneurysm cavity. The channel flow was circulated using a pulsatile or continuous flow pump through tubes attached to the inlet and outlet. To avoid the impedance mismatch between the other side of the channel wall and the air, the entire system was placed in a water container.

### Imaging pipeline

The imaging process began with an inverted microscope, which transmitted live images to our processing pipeline (Fig. 2a). We segmented the initial image into channels and obstacles using the segment anything model^56^, chosen for its ability to accurately differentiate complex visual elements. Following segmentation, we refined and cleaned the image with a morphological closing operation and adaptive thresholding to identify microrobots, which appeared black under the microscope. We then applied detection and tracking algorithms to identify the agent (microrobot), calculate the centre of the microrobot, plot a bounding box around it and initialize the channel and spatial reliability tracker (CSRT), which was selected due to its robust tracking capabilities in dynamic and cluttered environments. When tracking was lost, the system quickly re-detected and initiated tracking, thus minimizing computation and enhancing real-time feedback. In the processed images, microrobots are marked in blue and the target locations in red. Positive rewards were assigned when the microrobot progressed towards the target, whereas movement away incurred a negative penalty.

### Reward function

This simulated setting enabled us to refine and iterate our reward functions and control strategies efficiently, without the continuous need for live experimental adjustments. This approach streamlines the development process, facilitating more precise and effective advances in microrobot control. The reward function is designed to incentivize the microrobot to efficiently reach designated target points while navigating around obstacles and taking into account various shapes and layouts of the channels. Formally, the reward *R* at time step *t* is defined by the following criteria:

$${R}_{t}=\begin{cases}\alpha, & \text{if target reached,}\\ -\beta, & \text{if a collision occurs,}\\ -\gamma f\left({d}_{t}\right), & {\rm{otherwise,}}\end{cases}$$

where *α*, *β* and *γ* are coefficients that weight the importance of each component in the reward function. The term *d*_*t*_ denotes the Euclidean distance to the target point at time step *t*, and *f*(*x*) = 1/(*d* + *ε*) is a real, monotonic function that translates the distance into a penalty (or reward), where *ε* is a small positive constant used to avoid division by zero. This function was specifically chosen to inversely relate the reward to the distance, thereby encouraging the microrobot to minimize this distance (Supplementary Note 2).

After extensive experimentation, we identified the optimal settings for our system: *α* = 10, *β* = 2 and *γ* = 0.1. Our simulation results confirm that MBRL effectively learns advanced navigation tactics through interactions within the environment. Thereby, it can master complex navigational strategies in intricate settings such as vascular systems, mazes and racetracks.

The adapted reward function for the flow environment *f*(*d*_*t*_, $\mathbf{X}_t,\mathbf{A}_t$) is defined as follows:

$$\begin{array}{l}f\left({d}_{t},\mathbf{X}_t,\mathbf{A}_t\right)\\=\begin{cases}-\mu, & {\rm{if}}\;\mathbf{X}_t\;{\rm{is}}\; {\rm{on}}\; {\rm{the}}\; {\rm{wall}}\; {\rm{and}}\;\mathbf{A}_t\;{\rm{is}}\; {\rm{in}}\; {\rm{the}}\; {\rm{direction}}\; {\rm{of}}\; {\rm{the}}\; {\rm{wall}},\\ -\kappa, & {\rm{if}}\;\mathbf{X}_t\;{\rm{is}}\; {\rm{central}}\; {\rm{in}}\; {\rm{the}}\; {\rm{channel}},\\ \displaystyle\frac{1}{d+\epsilon }-\lambda, & {\rm{otherwise.}}\end{cases}\end{array}$$

The components of the reward function are initialized as follows. A step penalty *λ* is applied at each step to encourage the microrobot to reach the target quickly. The wall sliding penalty −*μ* is imposed when the microrobot is in contact with a wall and the action taken is in the direction of the wall, allowing for sliding along the wall but discouraging pushing against it. The inverse distance reward 1/(*d* + *ε*) provides a continuous incentive for the microrobot to move closer to the target, with stronger gradients as the distance decreases. Moreover, we introduced a centring penalty −*κ* for when the microbot is too centrally located in the channel, which encourages the microrobot to stay near the walls where the drag forces are lower. These adjustments incentivized the microrobots to navigate closer to the channel walls, where drag forces are substantially reduced due to the no-slip condition.

### Training ratio

We investigated a critical parameter known as the training ratio, which denotes the number of steps trained in the imagination (within the world model) relative to each step in the physical environment. This approach capitalizes on using the world model to simulate numerous hypothetical scenarios, thus reducing the need for extensive physical interactions. The key advantage of a higher training ratio is its potential to enhance the efficiency of the learning process. It enables the agent to learn from imagined experiences, which are both quicker and less costly in terms of imagination than physical interactions. Ideally, using a higher training ratio reduces the number of environmental interactions required to achieve convergence.

We experimented with various training ratios to assess their impact on learning efficiency and performance. For example, a training ratio of 10:1 means that for every experimental step, the agent performs ten steps in the dreamed environment. This strategy enables the agent to accumulate more experience and optimize its policy without the time and resource constraints associated with physical training. Conversely, a lower training ratio, such as 1:1, entails that the agent performs an equal number of physical and simulated steps, which slows down the learning process but provides more accurate feedback from the physical environment.

Our experiments demonstrated that higher training ratios, such as 1,000:1, dramatically reduced the number of interactions with the physical environment required to achieve convergence. The results indicate that higher ratios led to faster convergence, whereas lower ratios often failed to reach convergence. To maximize the benefits of high training ratios, we developed a parallel script to run physical environment interactions and world model training on separate threads. This resulted in an adaptive training ratio that was dynamically adjusted with the agent’s performance in the physical environment.

### RL implementation

We formalized the problem as a Markov decision process that includes the state space, action space, reward function and transition dynamics. The state, action and reward triplet at time *t* (*S*_*t*_, *A*_*t*_, *R*_*t*_) and the transition dynamics (*T*) enable the RL agent to learn optimal policies for microrobot control through continuous interaction with the environment. The state space *S*_*t*_ incorporates visual information captured by cameras, including the current position extracted from the image coordinates $({x}_{t}^\mathrm{a},{y}_{t}^\mathrm{a})$ and the target location coordinates $({x}_{t}^\mathrm{t},{y}_{t}^\mathrm{t})$, which represent the spatial location of the desired target position that the microrobot aims to reach:

$$\mathbf{S}_t=\left\{I,{x}_{t}^\mathrm{a},{y}_{t}^\mathrm{a},{x}_{t}^\mathrm{t},{y}_{t}^\mathrm{t}\right\},$$

where *I* encapsulates the processed camera feed at time *t*. We used a convolutional neural network to extract meaningful features from the images, such as the size, shape and interactions of the microrobots: *I* = CNN(Image_*t*_).

The action space *A*_*t*_ defines the set of all possible actions the control system can execute at any given time. In our settings, these actions pertain to the settings of the PZTs:

$$\mathbf{A}_t=\left[\left(\;{f}_{1},{A}_{1}\right),\left(\;{f}_{2},{A}_{2}\right),\ldots ,\left(\;{f}_{n},{A}_{n}\right)\right],$$

where *f* is the frequency of the ultrasonic travelling wave, *A* is the amplitude of the peak-to-peak voltage and *n* is number of transducers.

The transition dynamics *T*(*S*_*t*_, *A*_*t*_) describe how the state of the system changes in response to an action. This function is unknown to the RL algorithm and must be inferred through interactions with the environment. In our settings, the transition dynamics represent the physical changes in the system state resulting from an activated PZT:

$$\mathbf{S}_{t+1}=\mathbf{S}_t+\Delta t\times\text{dynamics}(\mathbf{S}_t,\mathbf{A}_t),$$

where Δ*t* is the time step, and dynamics(*S*_*t*_, *A*_*t*_) is a function modelling the physics of microrobot motion under ultrasound stimulation, which was extracted from the differences in the images (state).

### World model learning

The world model processes the state *S*_*t*_ into a latent state *Z*_*t*_ using an encoder–decoder architecture. This model predicts future latent states and rewards based on the current latent state and actions. It trains continually trains on new samples (*S*_*t*_, *A*_*t*_, *R*_*t*_). The key components include:

- Encoder–decoder architecture: This architecture compresses high-dimensional observations into a compact latent space for prediction and control. The encoder *q*_**ϕ**_ maps an observation *o*_*t*_ to a latent state *Z*_*t*_, where **ϕ** is a parameter vector shared between the encoder and all other world model components:
  $$\mathbf{z}_t \sim{q}_{\phi }(\mathbf{z}_t\mid\mathbf{h}_{t},{x}_{t}).$$
  The decoder (*D*) reconstructs the observation from the latent state: ${\hat{o}}_{t}=D(\mathbf{z}_{t})$.
- Dynamics network: This network predicts the future states of the microrobots based on their current state and actions, following the principle of a recurrent neural network. It preserves a deterministic state *h*_*t*_ predicted by the recurrent neural network using the previous actions *a*_*t*−1_, *h*_*t*−1_ and the previous embedded state *z*_*t*−1_:
  $$\mathbf{h}_t ={f}_{\phi }(\mathbf{h}_{t-1},\mathbf{z}_{t-1},\mathbf{a}_{t-1}).$$
- Reward predictor: This component predicts the rewards associated with different actions, aiding the agent in optimizing its behaviour. The reward predictor *R* estimates the reward *r*_*t*_ based on the latent state *z*_*t*_ and action *a*_*t*_:

$${\hat{r}}_{t} \sim{p}_{\phi }({\hat{r}}_{t}\mid\mathbf{h}_t,\mathbf{z}_t).$$

### Latent imagination and policy optimization

The agent generates future trajectories within the latent space and uses these imagined trajectories to train the policy and value networks. This reduces the need for physical interactions and makes learning more efficient. The main steps are as follows:

1. Trajectory sampling: Generate possible future trajectories by simulating the environment using the transition model (*h*_*t*_ = *f*_*φ*_(*h*_*t*−1_ | *z*_*t*−1_, *a*_*t*−1_). The imagined trajectories start at the true model states *s*_*t*_ drawn from the replay buffer of the agent and are then carried in the imagination by the transition model. These trajectories are generated much faster than the environment interaction and are controlled by a parameter called the training ratio. We developed a multi-threaded approach in which the latent model runs continuously on a separate process without a fixed ratio with the physical environment interactions.
2. Trajectory evaluation: Assess the quality of each trajectory based on the accumulated rewards predicted by the reward model. The reward predictor ($\hat{{r}_{t}}\sim{p}_{{{\phi }}}\left(\hat{{r}_{t}}\mid\mathbf{h}_{t},\mathbf{z}_{t}\right)$) estimates the rewards of each state.
3. Policy and value network training: The actor–critic component is trained to maximize the expected imagined reward $\left(E\left(\sum_{t=0}^{\infty }\gamma^{t}{r}_{t}\right)\right)$ with respect to a specific policy. The evaluated trajectories are used to update the policy and value networks, which dictate the agent’s actions in the physical environment.

This training loop leverages the predicted latent states and rewards, substantially enhancing sample efficiency by reducing the dependence on real-world interactions and relying on a very compact latent representation.

#### Algorithm 1

Microrobot MBRL training

**Require:** Configuration, frames, CSRT and segmented mask

**Ensure:** Environment set-up, reward calculation and state update

 1: **Initialize** environment with configuration parameters  # Set environment

 2: Initialize RL state *s*_0_  # Initialize RL state

 3: Downsize image to 64 × 64 px # Reduce image size

 4: **while** Episodes < Total_Episodes **do** # Main training loop

 5:  frame ← get_camera_frame;   # Capture frame

 6:  cleaned_frame ← segment_frame  # Segment frame

 7:  bubble_size ← detect_cluster # Detect cluster size

 8:  truncated, terminated ← False, False  # Initialize flags

 9:  **if** bubble_size > area_threshold **then** # Check bubble size

 10:   Track microrobot with CSRT # Track microrobot

 11:   agent_position ← get_agent_pos # Get agent position

 12   **if** agent_position ≈ target_position   # Near target

 13:   *r* ← Target reward, terminated ← True # Assign reward, end episode

 14:   **else if** agent_position in Channel_walls **then** # Collision detected

 15:   *r* ← Collision penalty, terminated ← True # Assign penalty, end episode

 16:   **end if**

 17:  **else**

 18:   *r* ← Distance_based reward # Distance reward

 19:  **end if**

 20:  **if** steps **>** threshold **then** # Check step limit

 21:   truncated ← True  # Mark truncated

 22:  **end if**

 23:  **Reset Collisions if necessary** # Reset if collisions

 24:  Deactivate PZT, adjust position and recheck collisions # Execute and assess action

 25:  **Apply Action, compute reward**

 26:  Execute action, observe environment and compute reward # Check and return results

 27:  Check termination, return (obs, reward, done)

 28: **end while**

#### Algorithm 2

Microrobot flow environment training and simulation

**Require:** Config, direction and amplitude

**Ensure:** Environment set-up, reward calculation and state update

 1: **Initialize** environment # Set up environment parameters

 2: Set **reward_centre** and **flow_direction** from config

 3: **if** reward_centre or flow_direction

 4:  Initialize **flow**  # Initialize flow

 5: **end if**

 6: reward ← 0  # Initialize reward for the step

 7: **if** is_valid_move (direction, amplitude) **then**  # Check if the move is valid

 8:  move_agent (direction, amplitude)   # Move the agent

 9: **else if** check_collision() **then**  # Check for collision

 10:  **if** is_valid_move (direction, amplitude/2) **then** # Try moving with reduced amplitude

 11:   move_agent (direction, amplitude/2)

 12:  **else**

 13:   reward ← reward_collision  # Apply collision penalty

 14:   update_radius() # Update the radius after collision

 15:  **end if**

 16: **else**

 17:  move_agent (direction, small_amplitude) # Move with a small amplitude

 18: **end if**

 19: **if** flow_active **then**  # Check if flow is active

 20:  **if** is_in_centre() **then** # Check if agent is in the centre

 21:   reward ← reward + reward_centre  # Add centre reward to total

 22:   **if** is_valid_move (direction, amplitude/1.5) **then** # Try to move against the flow

 23:   move_agent (flow_direction, amplitude)

 24:   **else**

 25:   update_radius () # Update radius if move is not valid

 26:   **end if**

 27:  **end if**   # Check step limit

 28: **end if**   # Mark truncated

 29: **Update step counters and check termination**

 30: increment_step_counter ()  # Increase the step counter

 31: **if** reached_target () **then**  # Check if target is reached

 32:  reward ← reward_target_reached  # Add target reached reward

 33:  mark_as_terminated ()  # Mark episode as terminated

 34: **else if** radius_too_small () **then**  # Check if radius is too small

 35:  reward ← reward_termination  # Add termination reward

 36:  reset_radius()  # Reset radius for new episode

 37: **else**

 38:  reward ← calculate_distance_reward () # Calculate reward based on distance

 39: **end if**

 40: **Return observations, reward, done and info**  # Return step results

 41: **return** get_observations(), reward, is_done(), get_info()

## Supplementary information

Supplementary Information Supplementary Notes 1–13, Figs. 1–17 and legends for videos 1–7.

Supplementary Video 1 Training process in MBRL with a real microfluidic racetrack channel. The left video shows early training (<100,000 steps) when the algorithm struggles to reach the target. On the right, after 340,000 steps, the algorithm shows improved target acquisition. The full process spanned 10 days due to real environmental interactions.

Supplementary Video 2 Transfer learning behaviour from a simulation environment to a real experimental environment of the same shape. The model converged in real experiments in just 3 h.

Supplementary Video 3 Continuous action training in a vascular channel. The left side shows blue tree branches in the rapidly exploring random tree star algorithm searching for the shortest path, marked in red when found. On the right, the microrobot is marked in blue and the next target in red. The video demonstrates microrobots attempting to follow the path in real time.

Supplementary Video 4 Transfer learning from a simulation environment to a real vascular channel using an MBRL model with sweeping actions.

Supplementary Video 5 MBRL general model trained on ten environments demonstrates its ability to perform across all ten environments and adapt to a new, unseen channel after just 30 min of extra training.

Supplementary Video 6 Autonomous manipulation in a flow environment after transfer learning from a simulation that mimics the flow, which guides the microrobot to move in a low-drag region near the wall.

Supplementary Video 7 Active and passive shapeshifting of a microrobot navigating obstacles in a microchannel. Passive deformation occurred when a single PZT was activated, whereas active manipulation involved dynamic shape changes using several PZTs for precise control and navigation.

## Data Availability

No public or pre-existing datasets were used in this study. All data generated during the study are available in the main text, the Supplementary Information and the repositories listed below. Simulation and physical training data, including reward trajectories, target success rates and trained model checkpoints for the racetrack, circuitous, multi-output, vascular, mixed and randomized environments, are available via Figshare at 10.6084/m9.figshare.28940828.v1 (ref. ^70^). Processed data from physical experiments, including microrobot trajectory metrics and performance evaluations across different environments, are provided as CSV files in the postprocessing directory of the GitHub repository (https://github.com/M-Medany/Model-Based-Reinforcement-Learning-for-Ultrasound-Driven-Autonomous-Microrobots). These files support the results shown in Figs. 3–6. Reward function definitions and training hyperparameters are provided in the Supplementary Information.

## Code availability

All data processing and analysis were performed using custom Python scripts. The source code used for the postprocessing, simulation training and microrobot control in the physical experiments is available via GitHub at https://github.com/M-Medany/Model-Based-Reinforcement-Learning-for-Ultrasound-Driven-Autonomous-Microrobots. An archived version of the code is also available via Zenodo at 10.5281/zenodo.15054076 (ref. ^71^).
