# Supplementary material for: Model-based reinforcement learning for ultrasound-driven autonomous microrobots
Source: Nat Mach Intell. 2025 Jun 26;7(7):1076–90. doi: 10.1038/s42256-025-01054-2 (PMC12283351; doi:10.1038/s42256-025-01054-2)
Supplement: Supplementary file 1 — Supplementary Notes 1–13, Figs. 1–17 and legends for videos 1–7. [file 42256_2025_1054_MOESM1_ESM.pdf]

nature machine intelligence

Supplementary information

https://doi.org/10.1038/s42256-025-01054-2

Model-based reinforcement learning for ultrasound-driven autonomous microrobots

In the format provided by the authors and unedited

Contents:

- Supplementary notes S1 to 13
- Supplementary Fig. 1 to 17
- Legends for movies S1 to S7
- SI References

Note S1. Experimental setup

The experimental setup for the autonomous manipulation of microrobots consists of three main components: the physical setup, imaging pipeline, and control unit.

1. Physical Setup

The physical setup includes a PDMS (polydimethylsiloxane) microfluidic channel, eight piezoelectric transducers (PZTs) as actuators, a function generator, and a microcontroller connected to an electronic circuit containing eight relays responsible for selectively activating and deactivating the PZTs. The function generator (Tektronix AFG3000) is used to generate the sinusoidal waves needed to manipulate the microbubbles. It allows for real-time modulation of acoustic signals, enabling precise control over frequency and amplitude. The microfluidic channels are fabricated using soft lithography techniques, beginning with a master mold patterned lithographically using SU-8 negative photoresist on a silicon wafer, which is then placed inside a Petri dish. The PDMS prepolymer is prepared by mixing the silicon elastomer base and curing agent in a 10:1 weight ratio, degassing the mixture under vacuum to remove air bubbles, pouring it into the mold, and curing it at 85°C for 2 hours. Once cured, the PDMS is peeled off the mold, and inlet and outlet ports are punched using a 1-mm punch. A separate, flat PDMS layer is created similarly using a blank wafer, with both layers cleaned using isopropyl alcohol and subjected to a 15-minute ultrasonic bath in water. After a 1-minute plasma treatment, the layers are aligned and pressed together at 85°C for 2 hours. Eight PZTs, each resonating at 2.8 MHz, are attached to the sides of the PDMS-embedded microfluidic channel using two-component epoxy glue.

The microbubbles used are ultrasound imaging contrast agents from Bracco Sonovue[1], which are prepared by injecting saline into a vial containing lyophilized sulfur hexafluoride lipid-type A powder and gently shaking the mixture to create stable microbubbles. These microbubbles have a sulfur hexafluoride gas core surrounded by a phospholipid monolayer shell that prevents coalescence, with sizes ranging from 2 to 9 µm. In our experimental setup, the concentration of these microbubbles directly influences the size and maneuverability of the microrobots. Higher concentrations cause the microbubbles to cluster more densely under ultrasound, forming larger microrobots that enhance collective acoustic responsiveness. This clustering is critical for dynamic control over the microrobot size and movement, optimizing their interaction with ultrasound waves for precise navigation and manipulation in microfluidic systems. To prevent standing wave formation and ensure traveling waves, the setup is submerged in water. The microbubbles remain evenly distributed without acoustic actuation, but applying an incident acoustic field causes them to aggregate into swarms due to the secondary Bjerknes force[2,3].

2. Imaging Pipeline

The imaging setup features a Canon EOS 6D Mark II camera mounted on a Leica DMI6000B inverted microscope, capturing live images that are transmitted via HDMI to the processing pipeline. A segmentation model identifies obstacles and navigable spaces within the microfluidic channel, with adaptive intensity thresholding used to detect microbubbles, which appear black under the microscope. The threshold is optimized through iterative trials, depending on camera settings and lighting conditions. Processed images highlight microbubble clusters in blue (0, 0, 255) and plot the current target location in red, matched to the cluster size. The segmentation model is specifically designed to differentiate the background from obstacles in the channel. This process begins with an operator-assisted segmentation model that separates the channel and obstacles (SAM)[4]. This model integrates a robust vision transformer with prompt-based interactions, delivering excellent zero-shot generalization and accurately segmenting new, unseen images without additional training, followed by a morphological closing operation using a (5, 5)

kernel over three iterations to refine the segmented mask, which is then applied to the original image. Morphological operations are useful for removing imperfections and smoothing the segmentation results. While different preprocessing methods like adaptive thresholding, Otsu's method, and edge detection techniques (e.g., Canny and Sobel) were explored, the best results were consistently achieved by feeding the raw image directly into the segmentation model. To enhance efficiency, segmentation masks for common channels are cached, reducing the need for repeated segmentation during restarts or resumptions, which significantly cuts down on computational overhead.

3. Control Unit

The control unit is a computer equipped with an NVIDIA RTX 4090 GPU with 24 GB of memory, running on Ubuntu. It oversees the MBRL training and manages the entire experimental setup. The control unit integrates data from both the physical setup (including the Arduino and function generator) and the imaging pipeline (capturing video signal from the camera via a capture card). It runs the control algorithms while dynamically adjusting experimental parameters, enabling real-time feedback and precise control. This integration is crucial for ensuring seamless coordination between the various components, allowing for efficient and responsive management of the experimental environment.

Note S2. Simulation environment and amplitude predictor.

Simulation Environment

We developed a simulation environment to model microrobot behaviour, intentionally excluding resonance and bubble size effects. This design decision was based on the simulation's purpose for training rather than replicating microrobots' complex dynamics. The theory was validated in a sample environment resembling the real setup to conserve resources and time. Our primary focus was on local path planning and obstacle avoidance, with the environment configured as a 64 x 64 RGB image, represented by gym.spaces.Box with uint8 values ranging from 0 to 255 and a shape of (64, 64, 3). Obstacles were delineated in black, the channel in white, target points in red, and the agent's position in blue. The agent's circular shape mimicked microbubble clusters observed in real-world scenarios **Supplementary Figure 1** . The simulation physics utilized the PyGame framework, providing high efficiency and easy integration with the MBRL environment. This simulator enabled rapid testing of various algorithms[5,6], architectures and parameters, facilitating quick iteration and development of control strategies.

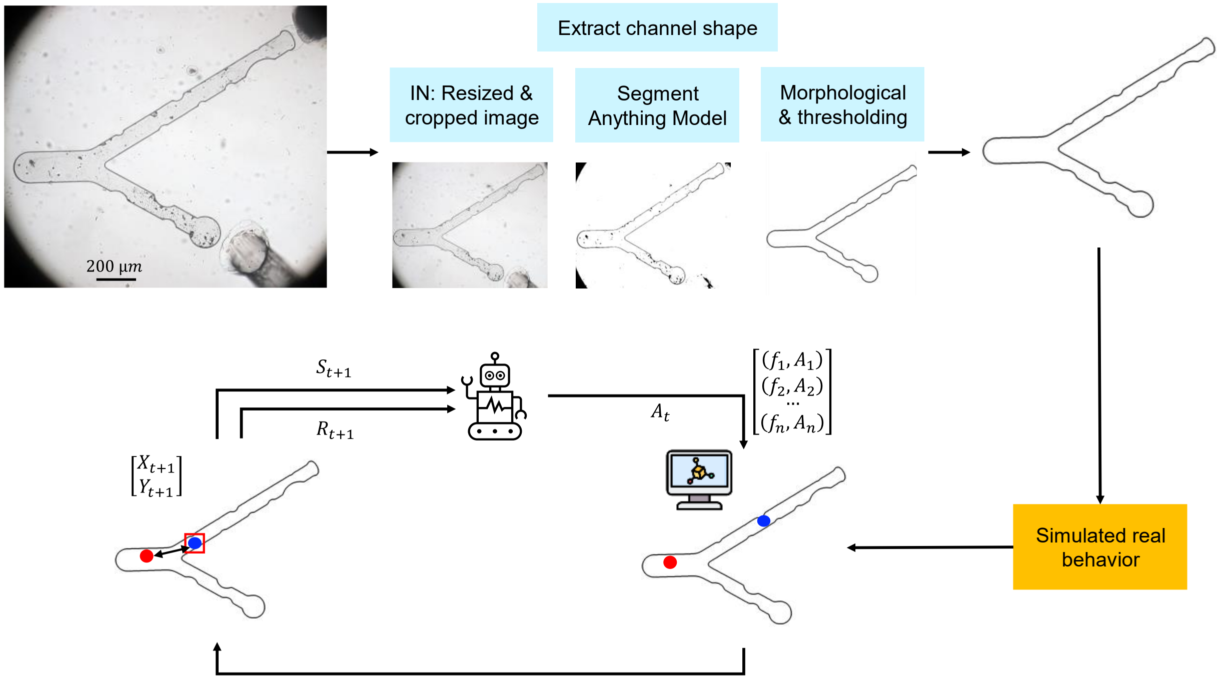

Supplementary Figure 1 | Simulation Environment: The process begins with extracting the channel shape from real microscope images (top left). The image is resized and cropped, followed by segmentation, morphological processing, and thresholding to identify the channel boundaries (top center). The extracted channel shape is used to create a simulated environment that mimics real-world behavior (middle right). The agent interacts with this simulated environment, receiving state updates and generating actions to navigate the channel (center). The simulator outputs the next state (Xt+1, Yt+1) based on the agent's actions (bottom left) and updates the visual representation of the channel (bottom right). This iterative process ensures the simulated behavior aligns with real-world dynamics.

1. Reward Functions

We tested various reward functions to optimize microrobot behavior, balancing penalties for incorrect actions (e.g., wall collisions) and rewards for correct ones (e.g., reaching the target). The general reward function is:

$$R_t = \begin{cases} \alpha & \text{If target reached} \\ -\beta & \text{If collision occurs} \\ -\gamma .\ f(d_t) & \text{Otherwise} \end{cases}$$

where $f(d_t)$ is based on the distance to the target (d):

- **Binary Reward Function:** Provides fixed rewards for reaching the target (α = 10) and penalties for crashes ($\beta = 2$), with ($f(d_t) = 0$). This approach offers clear guidance but may struggle with long-horizon planning due to decaying rewards at larger distances.
- **Linear Distance Reward Function**: Rewards based on linear distance:
  $$f(d) = d + \upsilon \text{ where } (\upsilon)$$
  is a hyperparameter. It provides straightforward guidance but may suffer from diminishing gradients near the target.

- **Inverse Distance Reward Function**: Rewards inversely proportional to the distance:
  $$f(x) = \frac{1}{d + \epsilon}$$
  This function offers continuous feedback, increasing the pull towards the target as the distance decreases.
- **Logarithmic Distance Reward Function**: Uses a logarithmic scale:
  $$f(d) = \log\left(\frac{1}{d + \epsilon}\right)$$
  It offers strong gradients near the target, stabilizing navigation and preventing overshooting.
- **Squared Distance Reward Function**: Employs an inverse quadratic scale:
  $$f(x) = \frac{1}{(d + \epsilon)^2}$$
  This function intensifies the reward as distance decreases, driving precise movement towards the target, but requires careful tuning to ensure stability.

2. No Collision

To further refine the reward structure, especially for environments where microrobots slide along walls, we introduced the "No Collision" reward function. This modified function removes harsh penalties for collisions and instead implements small penalties and step rewards to encourage effective navigation while allowing flexibility in handling obstacles.

The reward function is defined as follows:

$$R_t = \begin{cases} \alpha & \text{If target reached} \\ -\gamma .\ f(d_t, X_t, A_t) & \text{Otherwise} \end{cases}$$

Where: $f(d_t, X_t, A_t) = \begin{cases} -\mu & \text{If } X_t \text{ is on the wall and } A_t \text{ is in the direction of the wall} \\ \frac{1}{d+\epsilon} - \lambda & \text{Otherwise} \end{cases}$

- **Positive reward for reaching the target** (α): Fixed positive reward for reaching the target.
- **Wall sliding penalty** (μ): Small penalty when the microrobot is in contact with a wall and the action taken is in the direction of the wall, allowing sliding but discouraging pushing against the wall.

- **Inverse Distance Reward** $\left(\frac{1}{d+\epsilon}\right)$: Continuous incentive for the microrobot to move closer to the target, providing stronger gradients as the distance decreases.
- **Small step penalty** (λ): This constant penalty encourages the microrobot to minimize the number of steps taken to reach the target. It is designed to keep the reward always negative.

These modifications ensure that the microrobot is able to handle collisions gracefully by allowing sliding along walls: it can adapt its shape and path more naturally in response to the fluidic environment.

It is important to note that with this reward function, the cumulative reward for a long episode where the target is not reached can become very negative. This occurs because the reward is always negative until the target is reached, accumulating penalties over time. In contrast, the previous function reset the episode upon collision, incurring only a fixed penalty of -2.

Amplitude Prediction

We developed an amplitude predictor to reduce the action space by determining the optimal amplitude for the function generator based on the observed area of the micro-robots. The relationship between bubble size and required amplitude was modelled using experimental data. The amplitude predictor uses a formula correlating the observed area of the microbubbles to the required voltage:

$$A = k \cdot \sqrt{area} + b$$

where A is the amplitude in volts, area represents the area of the microbubbles in pixels, and k and b are empirically determined calibration constants. This approach allows the amplitude to vary from approximately 4 volts for very small bubbles to 18 volts for larger clusters.

Integrating the amplitude predictor reduced the action space from 64 to 16 actions, optimizing control strategy and improving training efficiency without compromising control over the micro-robot's velocity and stability.

Note S3. Transfer learning from simulation to real environment

Pre-training in a Racetrack Channel

The pre-training phase involves training the reinforcement learning (RL) algorithm within a simulated environment specifically designed to mimic the real-world conditions of microrobot navigation. To ensure the simulation closely matches the experimental setup, we captured images from the physical environment, segmented them, and used them to create the simulation environment. Additionally, we ensured that the actuator positions in the simulation were aligned with the experimental setup. For example, in the four-channel racetrack, we positioned the four PZTs in the simulation to match the experimental configuration.

The following adjustments were made to enhance the simulation:

- **Enhanced Collision Physics Simulation**: The simulator incorporates a more realistic collision model. In this model, when microrobots contact the channel walls, they flatten slightly, simulating the physical deformation that occurs in real scenarios. This enhancement provides a more accurate representation of the microrobots' behaviour upon impact, improving the reliability of the simulation.
- **Size Randomization**: To mirror real-world variability and prevent the model from overfitting to a specific microrobot size, the simulator randomizes the microrobot size at the beginning of each episode. This ensures that the model is exposed to a range of sizes, promoting generalization across different scenarios.
- **Speed Randomization**: The simulator also introduces variability in the agent's speed at each instant, which is inversely correlated with the frequency of specific actions. This approach encourages the exploration of less frequently used actions, avoiding the model's collapse into a single dominant behaviour. The speed in each direction is sampled as a normal random variable, where the mean is inversely proportional to the soft max of the action frequencies:

$$s_d \sim \mathcal{N}\left(\sigma\left(\frac{1}{f_i}\right)\right), \theta^2)$$

  Where:

  - s_d is the speed in direction d.
  - f_i is the frequency of action iii in the "actions-buffer" (a storage of the most frequent actions).
  - $\sigma\ f_i$ is the softmax function applied to the frequency f_i, which converts these frequencies into a probability distribution:

$$\sigma(f_i) = \frac{e^{f\,i}}{\sum_{j=1}^{k} e^{f\,i}} \quad \text{for } i = 1,2, \ldots., k$$

  - K is the number of possible directions.
  - θ^2 is the variance of the speed, controlling the spread of the normal distribution.

This method works by decreasing the speed in directions that are frequently chosen, thereby encouraging the exploration of less common actions. It helps to avoid overfitting to a specific set of behaviors and increases the entropy (randomness) of the action space, leading to more robust learning across a broader range of conditions.

Once the model achieves satisfactory performance in the simulation environment, it is transferred to the real experimental setup. To ensure a smooth transition, the final portion of the replay buffer from the simulation is also transferred, bypassing the initial exploration phase in which the RL algorithm would otherwise fill the buffer with random policies. During the initial adaptation phase, we observe a brief performance degradation due to the domain shift between the simulation and real environments, as shown in **Supplementary Figure 2a**. However, this adaptation period is short, and the model quickly adjusts to real-world conditions, effectively bridging the domain gap to reach the target, as shown in **Supplementary Figure 2b**. Importantly, the pre-trained model shows significantly better performance compared to a model trained from scratch in the real environment.

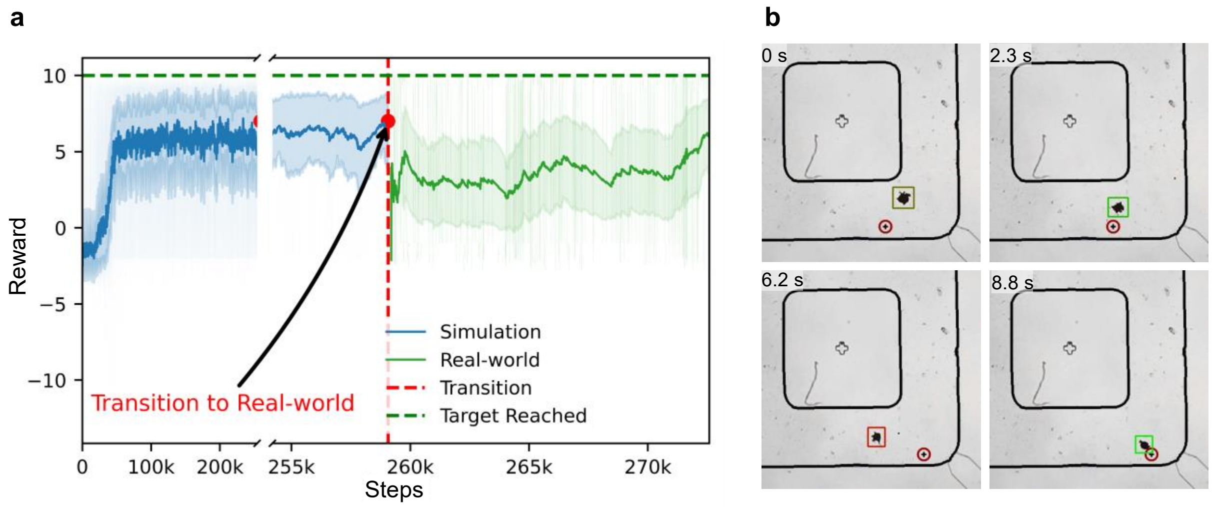

Supplementary Figure 2 | Transfer learning in a racetrack channel. a. A plot of RL performance illustrates the transition of a pre-trained model from simulation to the real-world environment. The score rapidly increases during the simulation phase (blue), drops after the transition to real-world experiments (marked by a red vertical dashed line), and then rises again as the model adapts to the real-world environment (green). Solid lines represent the exponentially weighted moving average (EWMA) of the reward ($\alpha = 0.01$), and shaded regions indicate ±½ of the rolling standard deviation (window = 50). **b.** An image sequence demonstrating a microrobot navigating an artificial racetrack channel, starting from the initial position and sequentially reaching the target. A red box marks the microrobot when it is far from the target, while green indicates proximity to the target. The target position is denoted by a circle with a black plus symbol.

Note S4. Continuous action space implementation

Initially, our simulation used discrete actions, as only one piezoelectric element was activated at a time, each with a specific frequency and amplitude, resulting in 64 possible action combinations. However, when transitioning to the experimental setup, our goal was to optimize frequency and amplitude as continuous actions. To simplify this problem, we manually provided actuation corresponding to discrete actions and used RRT* for path planning. This approach allowed us to efficiently navigate the continuous action space while maintaining control precision.

During training, frequent adjustments in frequency led to overshooting targets, causing instability in microrobot movements. The model often selected higher amplitudes to reach targets more quickly, which initially appeared beneficial but ultimately increased instability. The dynamic nature of the microrobot's environment and the complexity of the action space presented significant challenges in achieving stable and reliable performance. Despite these challenges, employing continuous actions and path planning allowed for successful navigation of the microrobots as shown in **Supplementary Figure 3**. However, frequent oscillations in frequency and amplitude compromised precise control, hindering the ability to follow designated paths accurately.

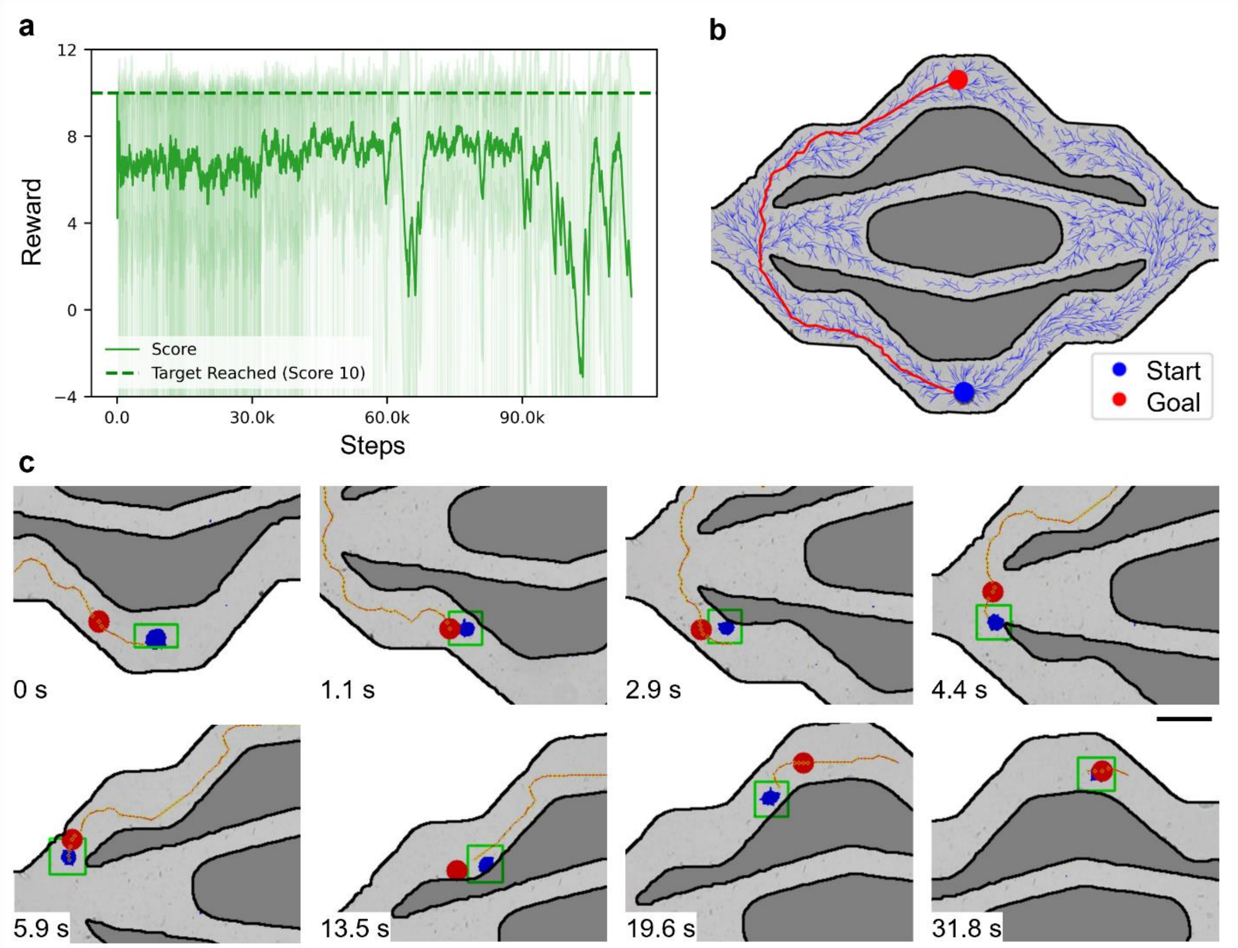

Supplementary Figure 3 | Training with Continuous Actions. a. A plot displaying the relationship between the reward and the steps , illustrating the training progress and performance trends. The solid line represents the exponentially weighted moving average (EWMA) of the reward ($\alpha = 0.01$), and the shaded region shows ±½ of the rolling standard deviation (window = 50). **b.** RRT* path planning within an artificial vascular channel. **c.** A sequence of images showing the microrobot (blue) following the preplanned path (yellow) to reach updated targets (red). The updated targets and intermediate points are marked along the path to visualize the microrobot's tracking accuracy and performance.

Another key limitation of this approach is the inefficiency of RRT* planning: once a path ended, significant time was required to calculate a new path. While effective in static conditions, it proved too slow to react to dynamic changes, such as fluid flow. We also explored a hybrid action space, combining discrete actions for piezoelectric activation with continuous actions for frequency and amplitude modulation. However, this approach significantly increased experimental time, making it impractical for our purposes. We opted for RRT* to streamline the process and ensure efficient and effective path planning.

Note S5. Flow simulation adjustments

Several adjustments have been made to enhance the robustness and adaptability of the microrobots in dynamic flow conditions:

- **Reward Function Adjustments**: To encourage microrobots to avoid areas with high drag forces, we adjusted the reward function to impose penalties for microrobots moving in the centre of the channel. This adaptation incentivizes the microrobots to navigate closer to the channel walls, where the drag forces are significantly reduced due to the no-slip condition.

  The adapted reward function $f(d_t, X_t, A_t)$ is defined as follows

$$f(d_t, X_t, A_t) = \begin{cases} -\mu & \text{If } X_t \text{ is on the wall and } A_t \text{ is in the direction of the wall} \\ -\kappa & \text{If } X_t \text{ is central in the channel} \\ \dfrac{1}{d+\epsilon} - \lambda & \text{Otherwise} \end{cases}$$

  Where we added a Centring Penalty (κ): A penalty for being too centrally located in the channel, encouraging the microrobot to stay near the walls where drag forces are lower.

- **Physical Model Refinements**: We refined the physical model of microbubble dynamics to better simulate bubble-wall interactions. The second Bjerknes force, which induces attraction and subsequent adhesion of microbubble clusters to the channel walls, was incorporated into the MBRL model. This interaction results in the formation of a "mirrored cluster" effect, allowing microrobots to leverage the reduced shear forces near the walls for easier movement.

- **Simulated Flow Force**: To realistically simulate the physical challenges encountered by microrobots in flow conditions, we introduced a simulated force that continuously pushes against the microrobots. This force is quantified in pixel values corresponding to the flow rate we aim to counteract. By simulating this dynamic force, the microrobots can adapt their strategies to navigate effectively within the flow. Furthermore, this force is stronger the more the bubble is in the center of the channel, behaving according to the Bernoulli equation.

Note S6. Dynamic Ultrasound Shape-Shifting Microrobots

We have developed and automated a shape-shifting strategy that enables microrobots to adapt dynamically when encountering obstacles (**Supplementary Figure 4a**). The process begins with an image-processing pipeline that utilizes the SAM for precise segmentation of the microchannel and microrobot (**Supplementary Figure 4b**). This pipeline detects the narrowest point in the channel and identifies it as an obstacle, guiding the microrobot to approach and cross it while navigating toward the target (**Supplementary Figure 4c**). **Supplementary Figure 4d** shows the microrobot dynamically deforming to cross the obstacle, and
Supplementary Figure 4e illustrates its movement towards the target after successful crossing.

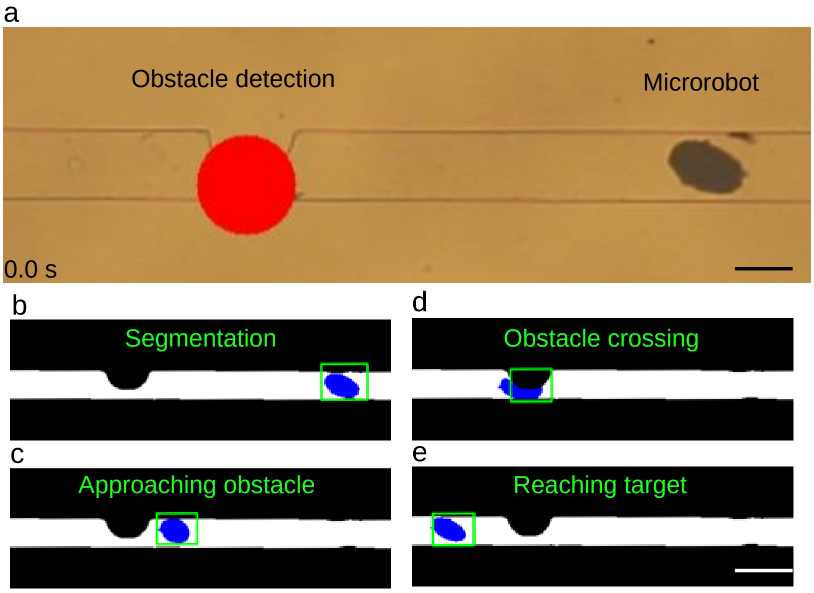

Supplementary Figure 4 | Dynamic Obstacle Navigation of Ultrasound-Driven Microrobots: a Microfluidic channel with a red-labeled obstacle, captured under an inverted microscope. **b** Segmented image using SAM: the microrobot is shown in blue and the tracker in green. **c** Detection of obstacle proximity triggers activation of Y-axis PZTs for shape adaptation. **d** The microrobot dynamically deforms to navigate around the obstacle. **e** Following obstacle crossing, the microrobot adjusts its trajectory to reach the target on the left.

Experimental Setup and Microrobot Manipulation: Our experimental setup consists of four PZTs arranged in a square configuration (**Supplementary Figure 5a**) bonded on the sides of a PDMS-based microchannel. This configuration enables precise ultrasound control of microrobot motion and shape.

Dynamic Deformation for Passive Deformation: Upon exposure to ultrasound, the microrobots self-assemble into a larger circular cluster, consisting of individual microbubbles ranging from 2 to 5 microns in size. Activation of PZT 4 induces left-to-right movement of the microrobots within the channel due to the primary radiation force. When encountering a semi-cylindrical obstacle positioned at the center of the channel, the microrobots undergo passive shape-shifting and squeeze through the orifice. This maneuver is facilitated by increasing the amplitude of the PZTs from 5 to 20 V_{PP}. **Supplementary Figure 5b** illustrates the repeatable right-to-left motion and squeezing of the microrobots through the obstacle by modulating PZT 2. However, this process requires higher voltages, which may lead to cavitation.

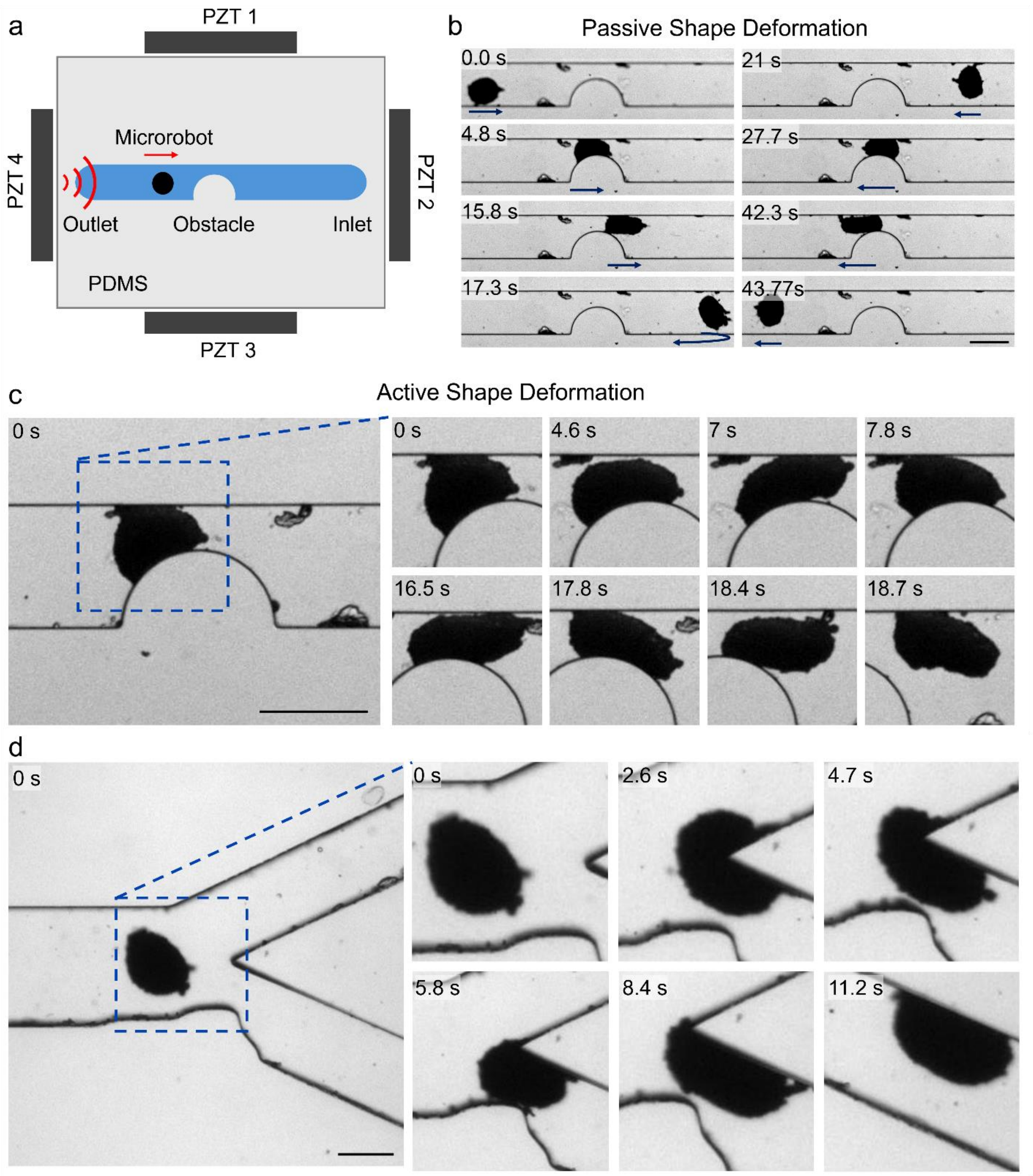

Supplementary Figure 5 | Microrobot Manipulation and Shape Deformation Demonstrated in Various Configurations: a. Schematic of the experimental setup, showing a straight microchannel equipped with four piezoelectric transducers (PZTs) positioned around an obstacle to manipulate the microrobot. **b.** Sequence of images illustrating passive microrobot deformation as it navigates through an obstacle using a single PZT activated along the X-axis at 20 volts, demonstrating the robot's ability to passively overcome the barrier. **c.** Series of images showing active manipulation where two PZTs on the Y-axis are activated, detailing the microrobot's dynamic shape deformation to navigate around the obstacle. **d.** Image sequence in a bifurcation channel setup where the microrobot encounters an obstacle on one side and actively shape-shifts to maneuver towards the downward path, showcasing advanced control and navigational capabilities. Scale bar: 100 µm.

Dynamic Deformation for Obstacle Navigation: Alternatively, we achieve dynamic deformation of the self-assembled microrobots. We first activate PZT 4 at 10 volts, propelling the microrobots from left to right. Upon encountering the semi-cylindrical obstacle, we simultaneously activate the Y-axis PZTs (PZT 1 and PZT 3) at an excitation frequency of 2.6 MHz with a lower amplitude of 5 volts. This induces controlled oscillations, allowing the microrobot to dynamically reshape and maneuver around the obstacle, as shown in **Supplementary Figure 5c**. The imbalance of forces from the transducers, caused by primary radiation forces acting on the robot and secondary radiation forces between the robot and the wall, results in dynamic deformation and simultaneous oscillation of the cluster at a frequency of 0.3 to 1.0 Hz. This approach reduces heat generation, making it appropriate for biomedical applications and enabling efficient navigation around obstacles.

Manipulation in Vascular Channels with Bifurcations: We further evaluated our microrobot manipulation strategy within a bifurcated, vascular-like channel featuring an embedded obstacle. The microrobots successfully adapted to the channel's geometry, navigating through the bottom branch and reshaping to fit within its confined space, utilizing similar ultrasound activation techniques as those used in dynamic deformation (**Supplementary Figure 5d**). This demonstrates the robustness of our control approach in realistic vascular environments.

Note S7. Microrobot Manipulation in 3D

Full 3D manipulation setups typically require side-view imaging for precise tracking of microrobots. In response to the reviewer's suggestion, we have demonstrated preliminary 3D control using an experimental setup featuring an array of 18 piezoelectric transducers placed in a conical channel, with coupling gel applied between the array and the microchannel for efficient ultrasound transmission (**Supplementary Figure 6a**). The 3D microchannel, where the microrobots are manipulated, is shown in **Supplementary Figure 6b**. The bottom view of the transducer array illustrates the arrangement of PZTs for 3D control (**Supplementary Figure 6c**). As the microrobots navigate through the 3D channel, changes in the focal plane cause them to appear blurry when moving out of focus. A sequence of images demonstrates the microrobot's movement in 3D as it leaves the focal plane, highlighting its ability to navigate beyond the primary field of view (**Supplementary Figure 6d**).

While this represents an important milestone, future work will focus on developing a full 3D setup that mimics in vivo vascular conditions by integrating multiple cameras at different planes for accurate tracking. Additionally, we aim to incorporate ultrasound imaging combined with AI-driven control to enhance microrobot manipulation in complex biological environments.

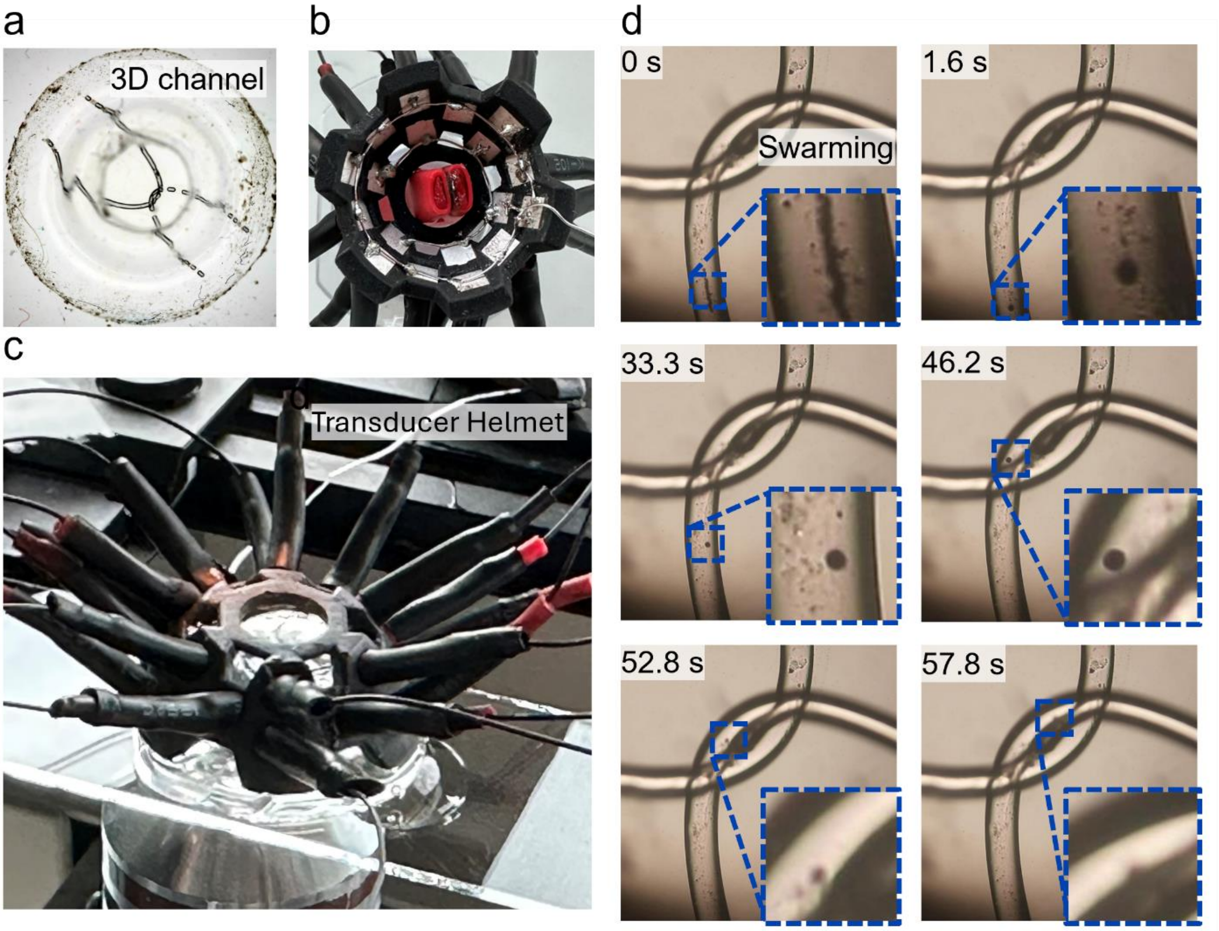

Supplementary Figure 6 | 3D Demonstration of Microrobot Manipulation: a. 3D microchannel where microrobots are manipulated. **b.** Bottom view of the transducer array, illustrating the arrangement of PZTs for 3D control. **c.** Experimental setup featuring an array of 18 piezoelectric transducers placed in a conical channel, with coupling gel applied between the array and the microchannel for efficient ultrasound transmission. **d.** Sequence of images showing microrobot movement in 3D as it leaves the focal plane, demonstrating its ability to navigate beyond the primary field of view.

Note S8. Hyperparameter PPO

we conducted hyperparameter search for PPO to ensure its implementation was fully optimized. We tuned several key hyperparameters, including the entropy coefficient (to balance exploration and exploitation), the value function loss coefficient (to stabilize training), the discount factor (to control the importance of future rewards), and the minibatch size and number of epochs (to optimize the use of rollouts). For the neural network architecture, we used the Nature CNN[7] to parameterize the policy and value function. These parameters were systematically varied to identify the optimal configuration for the task.

$Gamma$	$Minibatch\_size$	Num_epochs	vf_loss_coeff	$Entropy\_coeff$	$Reward$	$Rate\_target\_reached$
0.95	128	10	0.5	0.01	9.47 Â± 3.36	0.98 Â± 0.01
0.95	256	10	0.5	0.01	9.36 Â± 3.46	0.97 Â± 0.02
0.95	128	10	1	0.01	9.09 Â± 5.31	0.97 Â± 0.02
0.95	256	10	1	0.01	9.33 Â± 3.48	0.96 Â± 0.02
0.95	128	10	1	0.01	8.15 Â± 5.30	0.89 Â± 0.04
0.99	128	10	1	0.001	-6.70 Â± 19.81	0.55 Â± 0.08
0.99	128	10	1	0.01	-6.26 Â± 19.02	0.54 Â± 0.11
0.95	128	50	1	0.01	-6.14 Â± 14.79	0.44 Â± 0.06
0.95	256	50	1	0.01	-6.20 Â± 14.96	0.44 Â± 0.05
0.95	256	50	0.5	0.01	-6.19 Â± 14.56	0.43 Â± 0.05
0.95	128	50	0.5	0.01	-5.87 Â± 14.43	0.42 Â± 0.06

Supplementary Table 1: Hyperparameter Tuning Outcomes for PPO: This table displays the results from optimizing key hyperparameters of the Proximal Policy Optimization (PPO) algorithm, including the discount factor (Gamma), minibatch size, number of epochs, value function loss coefficient (vf_loss_coeff), and entropy coefficient (Entropy_coeff). Performance metrics, such as average reward and rate of target achievement, illustrate the impact of each configuration. Data are presented as mean ± standard deviation, highlighting the variability and effectiveness of PPO in different settings.

The results of the hyperparameter search are summarized in the table below. We observed that the combination of $Gamma = 0.95$, $Minibatch\_size = 128$, $Num\_epochs = 10$, $vf\_loss\_coeff = 0.5$, and $Entropy\_coeff = 0.01$ yielded the best performance, with a $Reward = 9.47 \pm 3.36$ and a $success\,rate = 0.98 \pm 0.01$. Other combinations, particularly those with higher discount factors (e.g., gamma = 0.99) or fewer epochs, resulted in significantly lower performance.

The impact of hyperparameter tuning on PPO's performance is further illustrated in Fig. 1, which shows the reward in **Supplementary Figure 7a** and the target success rate in **Supplementary Figure 7b** across different configurations. The plot highlights the significant improvement in performance achieved through tuning, as well as the variability in outcomes for different hyperparameter settings. Through this systematic exploration, we identified a robust set of hyperparameters for PPO, which allowed us to establish a strong baseline for comparison with the MBRL approach. Despite these optimizations, PPO still required approximately 20 times more steps than DreamerV3 to achieve comparable performance in complex environments, as shown in **Supplementary Figures 8 and 9**.

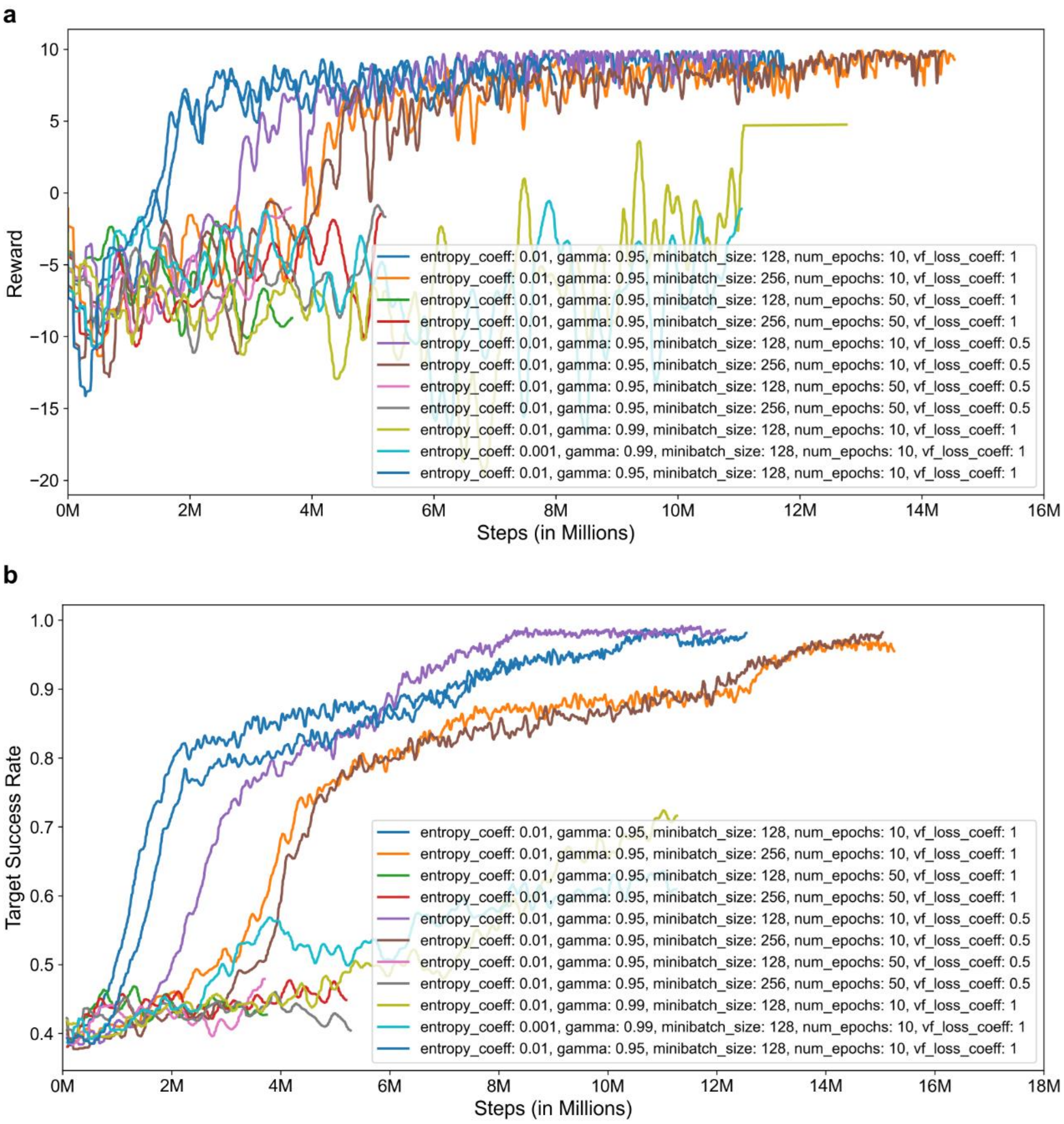

Supplementary Figure 7 | Hyperparameter Impact on PPO Performance. a. Reward trajectories over 16 million steps for various hyperparameter configurations, demonstrating their effect on rewards. **b.** Target success rates show the frequency of achieving desired outcomes under different settings, highlighting PPO's adaptability.

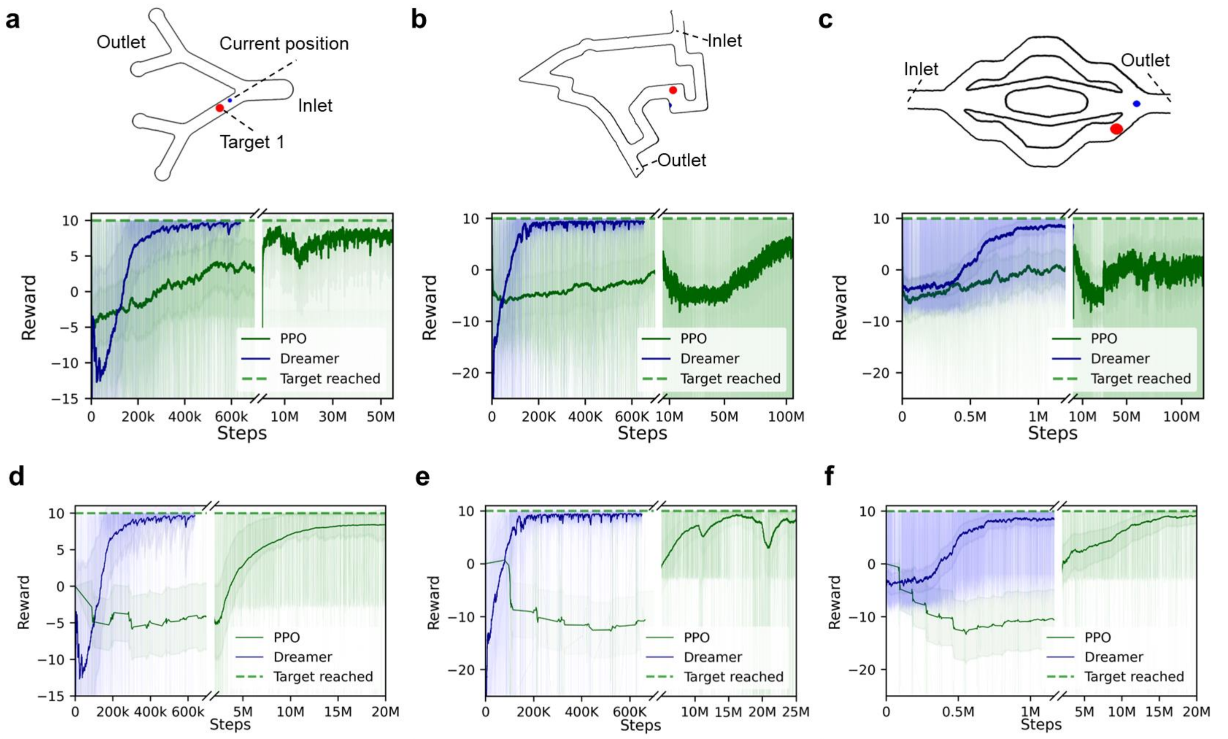

Supplementary Figures 8 | Comparative Analysis of Microrobot Navigation Performance in Diverse Channels. Reward trajectories illustrating the effectiveness of reinforcement learning algorithms in navigating: **a.** Multi-output Tributary channel, **b.** Circuitous channel, **c.** Vascular channels, with Dreamer V3 (blue) consistently outperforming the state-of-the-art PPO (green) across simulation steps. Further scenarios **d, e, f,** which involve post-hyperparameter tuning, demonstrate enhanced performance and adaptation to complex navigation tasks. Solid lines represent the exponentially weighted moving average (EWMA) of the reward, and shaded regions indicate ±⅓ of the standard deviation from the smoothed data ($\alpha = 0.002$). The dashed green line marks the success threshold (maximum reward of 10).

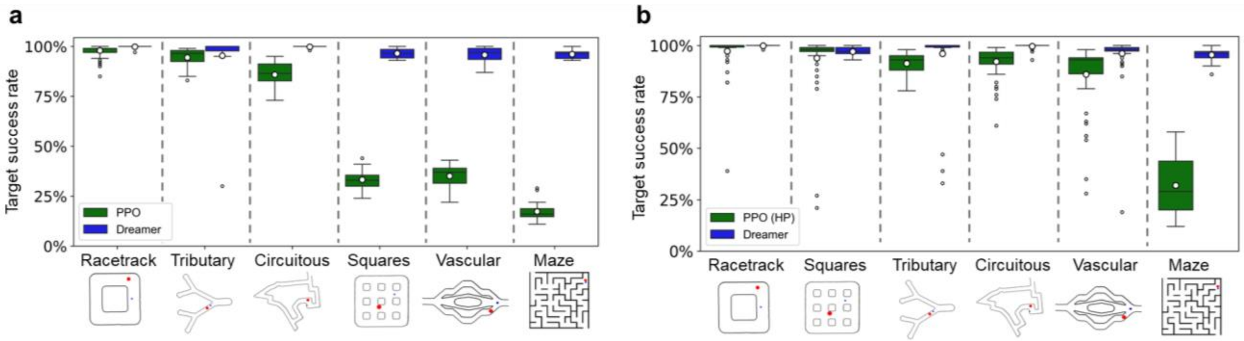

Supplementary Figures 9 | Algorithm Performance in Complex Navigation Scenarios. a. Comparison of PPO and Dreamer algorithms in reaching targets across different channel types: Racetrack, Tributary, SPA, Squares, Vascular, and Maze. **b.** Enhanced performance trajectories following extensive hyperparameter tuning, demonstrating improved efficacy and reliability in diverse environments. Box plots show the rate of target achievement evaluated over the final 50 episodes for each trained policy. Boxes represent the interquartile range (IQR; 25th–75th percentile), the central line indicates the median, whiskers extend to 1.5×IQR, and individual points show outliers. White circles denote the mean.

Note S9. Frame Skipping, Navigation Challenges, and Rescue Function in Microrobot Control

We evaluated different frame skipping rates (1, 2, and 4) during simulation to optimize training efficiency. A frame skipping rate of 4 significantly accelerated the simulation while maintaining acceptable performance. In real-world experiments, the microrobot operates at approximately 16 frames per second (fps). With a frame skipping rate of 4, each action is maintained for 0.25 seconds, which, in some cases, led to overshooting due to the prolonged action duration. To address this, we ensured that the tracking mechanism operated on every frame rather than strictly following the frame-skipping interval, improving overall robustness.

To further investigate the trade-off between simulation speed and real-world applicability, we tested a frame skipping rate of 8. While this further increased computational efficiency in simulation, it resulted in more significant overshooting during physical experiments. Based on these findings, we selected a frame skipping rate of 4 as the optimal balance between simulation speed and real-world performance, facilitating smoother transfer of the pretrained model to experiments. To illustrate the impact of frame skipping on learning performance, **Supplementary Figures 10.** presents a plot of accumulated rewards over training steps across different frame skipping rates (1, 2, 4, and 8). The results highlight how frame skipping influences learning stability and convergence rates.

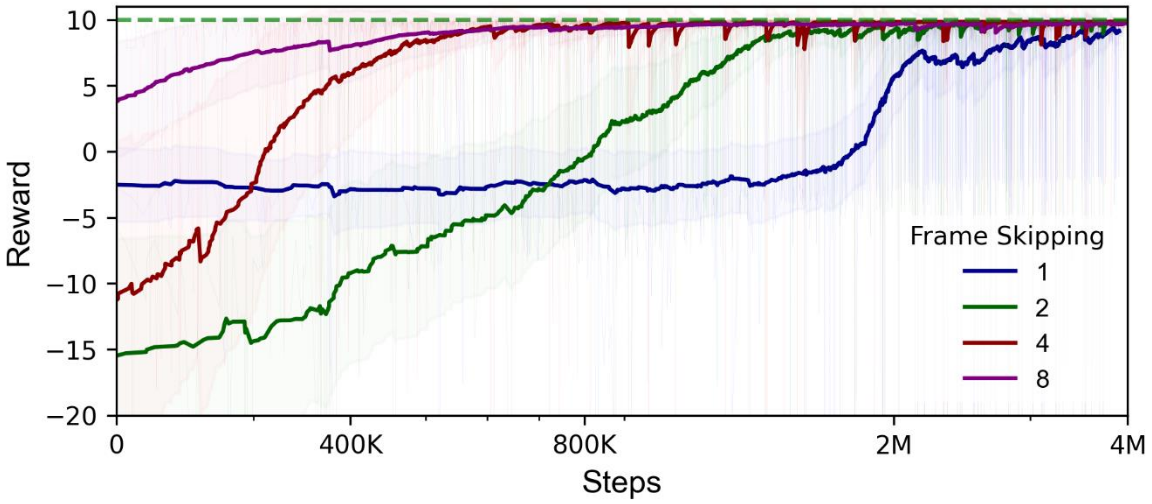

Supplementary Figures 10 | Impact of Frame Skipping on Learning Performance: This graph shows how different levels of frame skipping (1, 2, 4, 8 frames) influence reward accumulation over training steps, plotted on a logarithmic scale from 0 to 4 million steps. Solid lines show the exponentially weighted moving average (EWMA, $\alpha = 0.008$) of the reward, with shaded regions indicating ±⅓ of the exponentially weighted standard deviation. It demonstrates the varying rates at which the model learns and stabilizes, depending on the frame skipping setting.

Microrobot Navigation Challenges in Flow Conditions: As shown in **Supplementary Figures 11**, microrobot navigation under flow conditions presents significant challenges. In **Supplementary Figures 11a**, the microrobot cluster (blue) moves toward the target (red) within its tracking bounding box (green). However, as it reaches the end of the completed path, it begins to drift due to delays in generating a new path under flow (right to left). Similarly, in **Supplementary Figures 11b**, a new path is generated, but the microrobot's displacement due to the flow highlights the difficulty of maintaining control during real-time path planning. These observations underscore the importance of efficient path-planning strategies for microrobot stability.

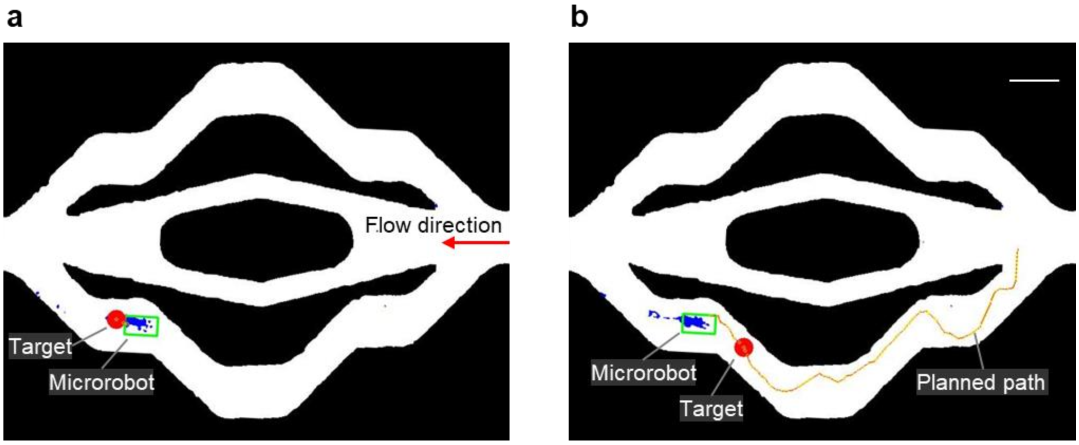

Supplementary Figures 11 | Challenges during microrobot navigation in flow conditions. a. The microrobot cluster (blue) with its tracking bounding box (green) navigating toward the target (red). As the microrobot reaches the end of the completed path under flow (right to left), it begins to be swept away due to delays in generating a new path. **b.** A new path is generated during the training process, but the microrobot's displacement caused by the flow highlights the challenge of maintaining control during real-time path planning. Scale bar: 300 µm.

Rescue Function for Microrobot Training and Recovery: To address challenges during training, we implemented a **rescue function** that intervenes when the microrobot overshoots its target, is lost due to high ultrasound amplitude or frame skipping, or deviates significantly from its intended path.

As shown in **Supplementary Figures 11**, the rescue function operates as follows:

- **(a) Overshooting:** The microrobot surpasses its target due to excessive ultrasound amplitude or extended frame skipping.
- **(b) Loss of Tracking:** The microrobot becomes undetectable, prompting the temporary shutdown of ultrasound actuation.
- **(c) Rescue Activation:** The last detected position of the microrobot is identified, its action is reversed to reposition it within the field, and tracking resumes. Training continues once the microrobot is successfully re-detected.

While the rescue function improves training stability, its effectiveness depends on accurate detection of the microrobot's last known position. **Supplementary Figures 12** further illustrates these recovery strategies, showcasing frame overlays of microrobot trajectories during overshooting, tracking loss, and the rescue process.

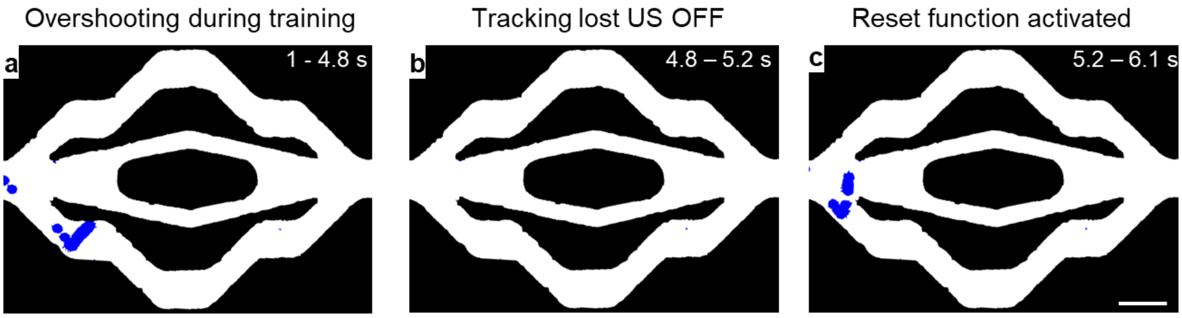

Supplementary Figures 12 | Challenges and recovery strategies during microrobot training. a. Overlay of frames showing the microrobot's trajectory as it overshoots its target during training due to high ultrasound amplitude or frame skipping. **b.** The microrobot becomes lost, leading to loss of tracking. In response, the ultrasound is turned off temporarily. **c.** Overlay of frames illustrating the microrobot's trajectory during the reset function. The microrobot's last detected position is located, its action is reversed to reposition it within the field, and tracking resumes. Training continues once the microrobot is successfully re-detected**.** Scale bar: 300 µm.

Note S10. Randomized environment and generalization

Enhancing Generalization: To test and enhance generalization, we initially demonstrated that training the model within a single environment, such as the four-output setup shown in (**Supplementary Figures 13**), results in a need for approximately 400,000 steps to adapt and achieve over a 90% success rate when transferred to a vascular environment. To improve upon this, we expanded the training across multiple simulated channels, exposing the model to a variety of channel shapes and obstacles. This extensive training quickly achieved a 90% success rate after approximately 50,000 training steps . For safety during fine-tuning, we take a snapshot of the new channel and adapt the model to it within these steps, ensuring it meets performance expectations efficiently. For example, in dynamically changing environments such as complex vasculature, the model is capable of training across the entire layout. As it moves through different parts, it is already adapted to those sections. However, if introduced to a completely different environment, further fine-tuning is necessary. This approach ensures that, particularly in biomedical applications, safety is prioritized to guarantee the model's reliability and effectiveness before transitioning to real-world experiments.

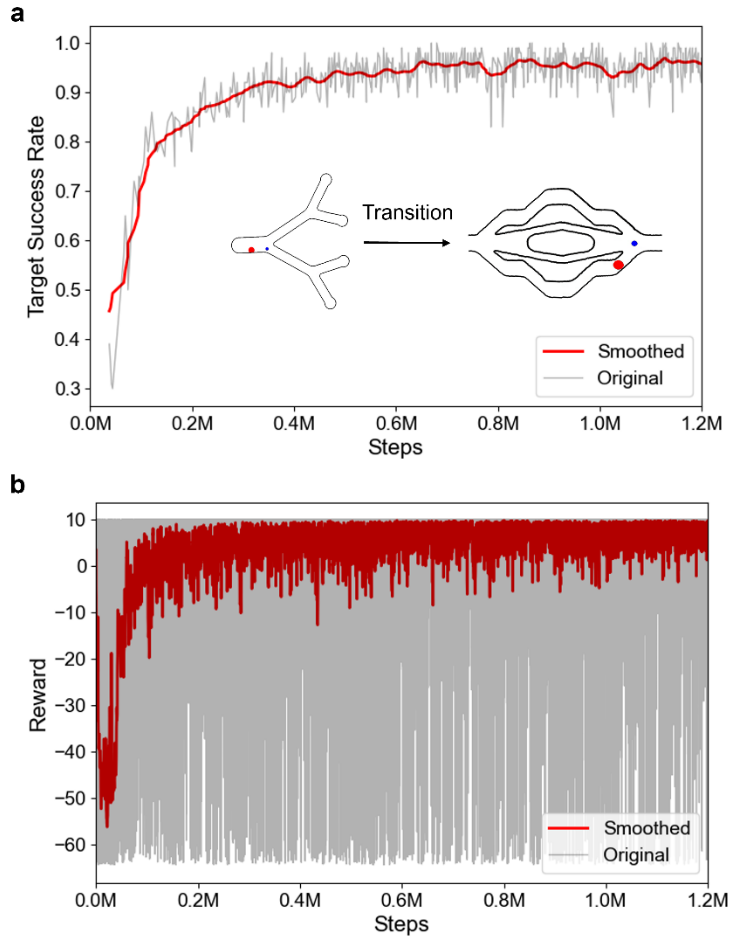

Supplementary Figures 13 | MBRL Model Transition from Multi-Output to Vascular Environment. a. Target success rate during fine-tuning from a multi-output environment to a vascular environment. The red line shows the smoothed curve using a Gaussian filter ($\sigma = 5$), and the gray line shows the original values. **b.** Reward trajectories during the same process, with the red overlay showing Gaussian-smoothed data and the gray curve representing raw rewards. In both plots, smoothing reduces high-frequency fluctuations to highlight learning trends, and the x-axis is scaled in millions of steps.

Randomized Environment: Building on our training strategy, which initially involved ten progressively challenging environments to showcase the model's adaptability, we integrated two additional randomized environments. These environments dynamically alter obstacle configurations by randomly switching white pixels to black, as illustrated in (**Supplementary Figures 14a**), generating unpredictable maps that significantly challenge the model. As training progresses, the complexity of these environments increases, with additional obstacles introduced to intensify the training challenge. After extending the training to 11 million steps, our model achieved a 70% success rate in navigating these complex scenarios, as demonstrated in (**Supplementary Figures 14b**), underscoring its enhanced adaptability and robustness. With the inclusion of additional channels and further randomization, we anticipate even higher success rates. However, considering that these microrobots are designed for use in biomedical applications within the vasculature, comprehensive pretraining is essential to meet all necessary safety measures and ensure the model's efficacy and reliability in clinical settings.

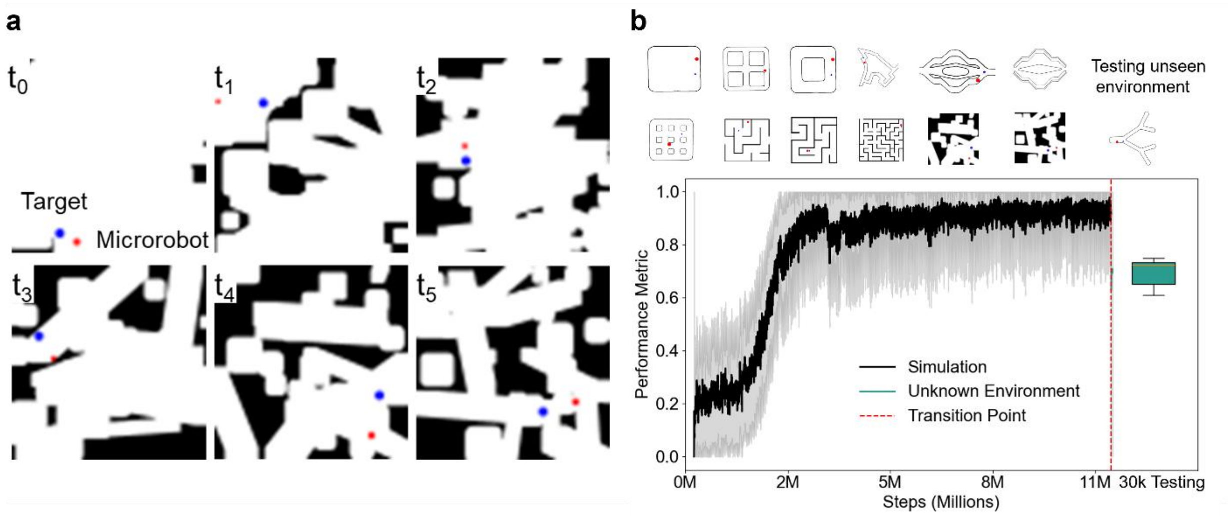

Supplementary Figures 14 | MBRL Model Training and Adaptation. a. Simulated Randomized Environment Complexity: Displays six images, each at a different time step, showing the increasing complexity of simulated randomized environments with obstacles (black) and paths (white). The microrobot (red) navigates towards the target (blue) through these evolving challenges. **b**. Generalization Across Environments: Depicts adaptation over 12 environments, including 10 original plus 2 newly introduced randomized environments, culminating in a 70% success rate in a novel testing environment, highlighting effective generalization. Solid lines show the exponentially weighted moving average (EWMA, $\alpha = 0.01$) of success rates, with shaded areas indicating ±½ of the rolling standard deviation (window = 50 steps). A red dashed line marks the environment transition. The final box plot shows the post-adaptation performance, with boxes representing the 25th–75th percentile and whiskers extending to 1.5×IQR.

Note S11. MBRL Implementation and world model learning

Definition of the World Model: The world model is a data-driven framework that learns the relationship between PZT inputs, microrobot motion, and environmental conditions (e.g., flow) directly from experimental and simulated data, without relying on explicit physics-based equations. The concept of a world model was first introduced in the influential *World Models* article[8], which proposed using a learned latent space to simulate environment dynamics and accelerate training. This idea was further refined in the *Dreamer V3* framework[5], where the world model serves as a recurrent neural network (RNN) that predicts future states, rewards, and observations based on past experiences and actions.

In our work, the world model captures the dynamics of microrobots under varying conditions, including PZT actuation, stagnant flow, and dynamic flow in channels of various complex geometries. It predicts microrobot motion (speed, orientation) and optimizes control actions to achieve task-specific goals (**Supplementary Figures 15**).

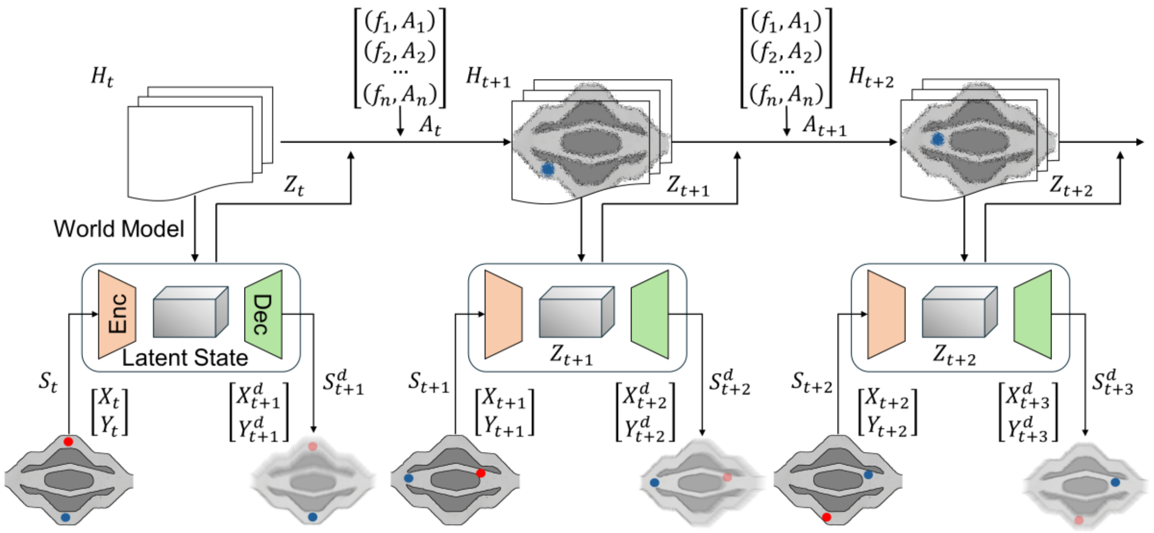

Supplementary Figures 15 | World Model Architecture: This figure illustrates the construction of the world model, which processes the state S_t into a latent state z_t using a Recurrent State-Space Model (RSSM). The model predicts future latent states and rewards based on the current latent state and input actions, effectively capturing the dynamics and critical features of the microrobot's environment. It continuously trains on new experimental samples (S_t, A_t, R_t), refining its predictions and adaptations over time. This detailed representation underscores the model's role in simulating and anticipating the outcomes of interactions within various settings.

World Model Learning

The world model processes the state S_t into a latent state Z_t using an encoder-decoder architecture. This model predicts future latent states and rewards based on the current latent state and actions. It continuously trains on new samples (S_t, A_t, R_t). The key components include:

- Encoder-Decoder Architecture: This architecture compresses high-dimensional observations into a compact latent space for prediction and control. The encoder q_ϕ maps an observation o_t to a latent state z_t, where ϕ is the shared parameter vector between the encoder and all other world model components:

$$z_t \sim q_\phi(z_t | h_t, x_t)$$

The decoder (D) reconstructs the observation from the latent state: $\hat{o}_t = D(z_t)$

- Dynamics Network: This network predicts the future states of the microrobots based on their current state and actions, following the principle of an Recurrent neural network (RNN). It preserves a deterministic state h_t predicted by the RNN using the previous actions a_{t-1}, h_{t-1}, and the previous embedded state z_{t-1}.

$$h_t = f_\phi(h_{t-1}, z_{t-1}, a_{t-1})$$

- Reward Predictor: This component predicts the rewards associated with different actions, aiding the agent in optimizing its behavior. The reward predictor R estimates the reward r_t based on the latent state z_t and action a_t.

$$\hat{r}_t \sim p_\phi(\hat{r}_t \mid h_t, z_t)$$

This training loop leverages the predicted latent states and rewards, significantly enhancing sample efficiency by reducing the dependence on real-world interactions and relying on a very compact latent representation.

Latent Imagination and Policy Optimization

The agent generates future trajectories within the latent space and uses these imagined trajectories for policy and value network training. This reduces the need for real-world interactions and allows for more efficient learning. The main steps involved are:

- Trajectory Sampling: Generating possible future trajectories by simulating the environment using the transition model ($h_t = f_\phi(h_{t-1}|z_{t-1}, a_{t-1})$. The imagined trajectories start at the true model states s_t drawn from the replay buffer of the Agent, then carried in imagination by the transition model. These trajectories are generated much faster than the environment interaction and are controlled by a parameter called "train-ratio". We developed a multi-threaded approach where the latent model runs continuously on a separate process without a fixed ratio with the real environment interactions.

- Trajectory Evaluation: Assessing the quality of each trajectory based on the accumulated rewards predicted by the reward model. The Reward predictor ($\hat{r}_t \sim p_\phi(\hat{r}_t|h_t, z_t)$) estimates the rewards of each state.

- Policy and Value Network Training: The Actor-Critic component is trained to maximize the expected imagined reward ($E(\sum_{t=0}^{\infty} \gamma^t r_t)$) with respect to a specific policy. The evaluated trajectories are used to update the policy and value networks, which dictate the agent's actions in the real environment (**Supplementary Figures 16**).

This training loop leverages the predicted latent states and rewards, significantly enhancing sample efficiency by reducing the dependence on real-world interactions and relying on a very compact latent representation.

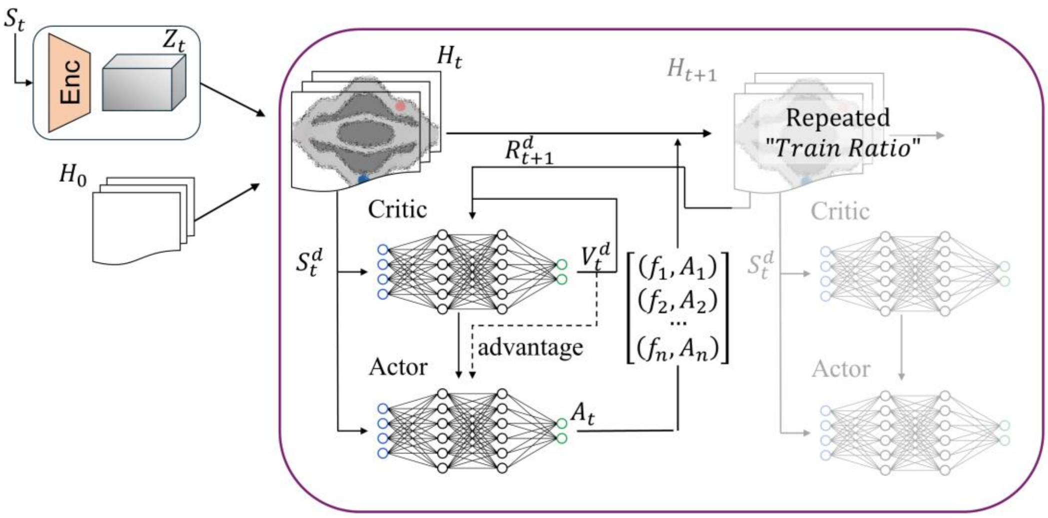

Supplementary Figures 16 | Actor-Critic Network: This diagram illustrates the integration of a Soft Actor-Critic Network with the World Model. The network is trained on latent observations Z_t derived from the state S_t by the encoder. It utilizes these observations to predict future states and rewards, employing a dual structure where the Critic evaluates the potential rewards and the Actor decides on the optimal actions. The process includes the use of a “Train Ratio” to repeatedly adjust the model’s exposure to training scenarios, enhancing its predictive accuracy and decision-making efficiency.

We utilize the Soft Actor-Critic (SAC) algorithm, which combines value-based and policy-based methods. It introduces entropy regularization to balance exploration and exploitation. The SAC training loop involves:

1. Updating the Policy Network: The actor (policy network) aims to select actions that maximize the overall reward while also exploring new possibilities. To achieve this, the policy network π_θ is updated to maximize the expected reward. An additional entropy term is included to encourage exploration by making the action distribution more diverse:

$$J_\pi(\theta) = \mathbb{E}[Q_\phi(s,a) - \alpha log\pi_\phi \theta(a \mid s)]$$

Here, $Q_\phi(s,a)$ represents the estimated return for taking action a in state s, and $\alpha log\pi_\phi \theta(a \mid s)$is the entropy term that ensures exploration.

2. Updating the Value Network: The critic (value network) aims to predict the return accurately. To do this, it minimizes the difference between its current predictions and the expected returns. This process is known as minimizing the Bellman residual:

$$\mathcal{J}_Q(\phi) = \mathbb{E}\left[(Q\phi(s,a) - (r + \gamma \mathbb{E}_{a' \sim \pi\theta}[Q_\phi(s',a')] - \alpha log\pi_\phi \theta(a' \mid s')))^2\right]$$

In this formula, r is the immediate reward, γ is the discount factor, and the term $\mathbb{E}_{a' \sim \pi\theta}[Q_\phi(s',a')$ represents the expected future rewards. The entropy term $\alpha log\pi_\phi \theta(a' \mid s')$ is included to maintain exploration.

3. Calculating Bootstrapped λ-Returns: To estimate long-term returns more effectively, we use bootstrapped λ-returns. This method combines immediate rewards with future value estimates:

$$\hat{R}_t \doteq r_t + \gamma v\phi((1-\lambda)v_t + \lambda \hat{R}_{t+1})$$

Here, λ is a weighting factor that balances between immediate and future rewards.

4. Learning the Critic Using Maximum Likelihood: The critic network is trained to predict the distribution of these bootstrapped returns by maximizing the likelihood of the observed returns:

$$L(v_\phi) = -\sum_{t=1}^{T} ln\, p_\phi(\hat{R}_t \mid s_t)$$

This objective function ensures that the predicted return distributions closely match the observed returns.

5. Adjusting the Entropy Coefficient: The entropy coefficient α helps balance exploration and exploitation. It is crucial to tune α appropriately depending on the reward scale and frequency in the environment. A higher α encourages more exploration, which is useful in environments with sparse rewards, while a lower α encourages exploitation, focusing on maximizing immediate rewards.

Microrobot Control: Linking PZT Inputs to Motion

Control Dynamics: When activated, piezoelectric transducers (PZTs) propel the microrobot almost perpendicularly away from the source due to acoustic radiation forces, which are proportional to the pressure gradient and the microrobot's volume. With eight PZTs arranged in orthogonal geometry, precise control over the microrobot's orientation and trajectory is achieved.

Adaptation to Action Space: Originally, our control model used discrete actions with four predefined frequencies, simplifying the initial training but limiting adaptability due to the variability in PZT responses and environmental interactions. To improve precision, we shifted to a continuous action space, allowing fine adjustments in frequency (2.7–2.9 MHz) and amplitude (4–14 V_{PP}). This transition, however, initially caused overshooting and erratic movements. To counteract these issues, we implemented sweeping actions around each PZT's resonant frequency, utilizing their inherent characteristics to stabilize motion, reduce overshooting, and ensure consistent performance at optimal frequencies.

Speed and Directional Control: The microrobot's speed is directly controlled by adjusting the voltage applied to the PZT. Higher voltages are necessary for larger microrobots due to increased drag, addressed by our amplitude predictor that calculates optimal voltage based on the microrobot's size using the formula: ($A = k \cdot \sqrt{area} + b$) where A is the amplitude in volts, area represents the area of the microbubbles in pixels, and k and b are empirically determined calibration constants. Directional control is achieved by activating specific PZTs, which produces predictable motion directions such as +X or -Y, validated through repeated testing.

Quantification, Training, and Real-Time Data Utilization: Microrobot speed and direction are quantified using image processing (OpenCV/Python), tracking trajectories frame-by-frame and calculating velocity as displacement over time. During training, the algorithm uses the microrobot's x and y coordinates to compute rewards—positive for reaching the target and negative for crashing. This reward mechanism guides the learning process, enabling the algorithm to optimize navigation strategies. During real-time experiments, the algorithm relies solely on image input for navigation, interpreting visual feedback to make control decisions. This simplifies the experimental setup and ensures robust, real-time performance as the model has learned to navigate based on visual cues. The world model is trained using a mix of simulated data from a custom Python simulator and experimental data from diverse channels and microrobot sizes.

World Model in Stagnant and Dynamic Flow Conditions

Stagnant Flow: In stagnant flow conditions, the environmental complexity is reduced, simplifying the navigation process towards the target. Inputs to the model include images that depict the microrobot's position, the target, and surrounding features. Control over the microrobot is achieved through discrete actuations of eight piezoelectric transducers (PZTs), which manage frequency, voltage, and transducer selection to automate trajectory adjustments.

- Reward Function: The model assigns positive rewards for reducing the distance to the target and negative rewards for inefficient movements or deviations from the target path.

- Training Process: Initially, the world model is trained on simulated data created in a gaming environment like PYGame. This foundational training is followed by experiments that test the microrobot's responses to PZT actuations in various real-world scenarios, accommodating different starting positions and microrobot sizes across consistent and varied channel geometries.

Dynamic Flow: Introduction of external flow adds complexity to the environment, influenced by factors such as drag forces, velocity gradients, and wall effects. The world model is adapted to account for these dynamics in its latent representations.

- Reward Function: The reward structure is adjusted to further incentivize progress towards the target and efficient navigation close to walls (where drag is minimal). Negative rewards are increased for collisions and movements into areas of high drag, encouraging the avoidance of such zones.

- Physical Principles: In dynamic settings, the model utilizes the no-slip condition to enhance microrobot maneuverability near walls and employs the secondary Bjerknes force to stabilize the microrobot, minimizing disruptions caused by surrounding fluid movements.

Note S12. Particle Image Velocimetry (PIV) Analysis

To accurately capture and analyze flow dynamics within the microchannel, we implemented Particle Image Velocimetry (PIV) techniques. Fluorescent microparticles (2 µm in diameter) were introduced into the flow to serve as tracers, allowing for precise visualization of flow behavior. **Supplementary Figures 17a** presents an overlay of these microparticles, illustrating their distribution and movement within the microchannel. For quantitative analysis, we processed the acquired PIV data using PIVlab[9], a robust tool for flow velocity evaluation. The resulting velocity profile is depicted in **Supplementary Figures 17b**, where a color gradient from red to blue represents the velocity spectrum red indicating maximum velocity and blue indicating minimum velocity. This methodical approach provides critical insights into the fluid dynamics affecting microrobot navigation and stability, enabling a comprehensive understanding of environmental conditions during experiments.

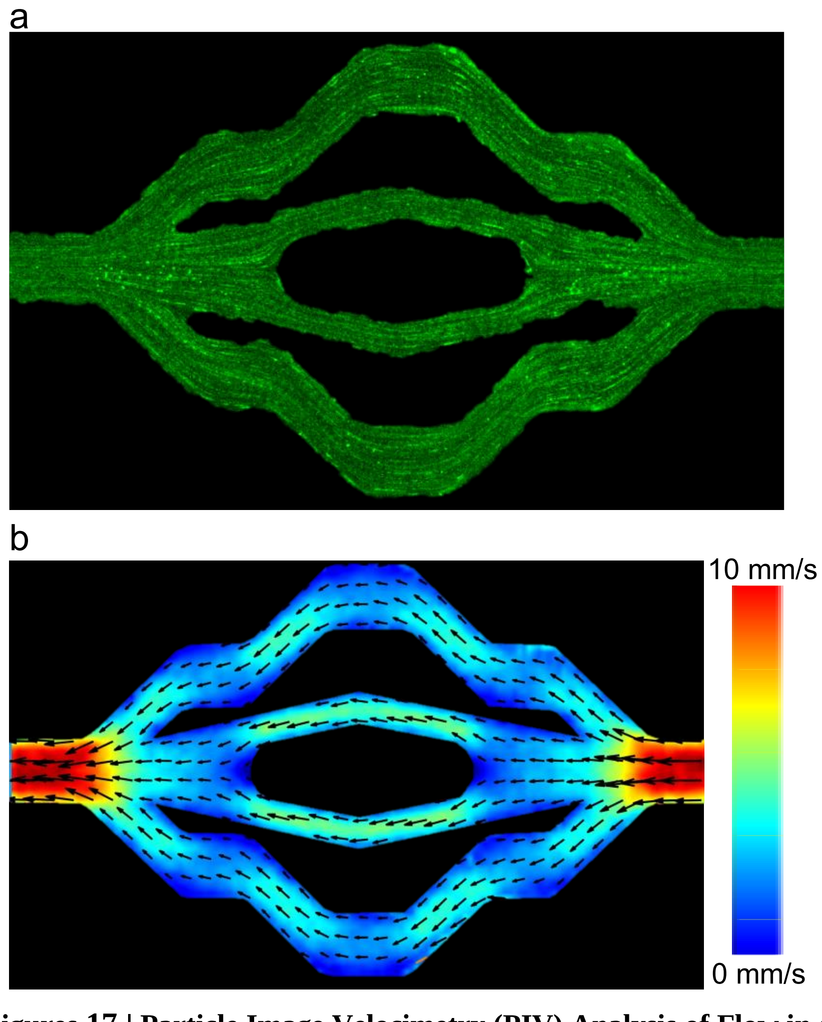

Supplementary Figures 17 | Particle Image Velocimetry (PIV) Analysis of Flow in a Microchannel. (a) Overlay of 2-µm fluorescent microparticles used to track flow dynamics. (b) Flow velocity profile analyzed using PIVlab, where the color gradient from red to blue represents maximum to minimum velocities, respectively.

Note S13. Algorithm for segmentation, reset function and frame skipping

Algorithm 1: Image Segmentation and Tracking Pipeline

Require: Input image from the camera
Ensure: Segmented image, Bounding box, and Initialized Tracker

```
Input: Resized & Cropped Image
segmented image ← SegmentAnythingModel(image)
morphed image ← MorphologicalCloseOperation(segmented image, kernel_size)
thresholded_image ← zeros_like(image)
thresholded_image[image ← threshold] ← [255, 0, 0]
cleaned_image ← thresholded_image AND morphed_image
bbox_size ←0
While bbox_size < min_cluster_size do
    bbox, bbox_size ← detect_cluster(cleaned_image)
End While
Tracker ← CSRT_tracker.init(morphed_image, bbox)
Output: Live Tracking, Location, Area
```

Algorithm 2: Collision Reset Function

```
for each step in Collision Reset Steps do                 ▷ Iterate through reset steps
    self.set_piezo_off()                                  ▷ Function generator off
    obs ← self.get_obs()                                  ▷ Iterate through reset steps
    pos ← self.tracker.get_agent_pos(obs)                 ▷ Get microrobot's position
    Count_collisions ← self.check_collisions (obs, pos, radius)
    piezo ←ARGMAX(count_collisions)                       ▷ Select piezo with most collisions
    self.set_piezo_on(piezo)                              ▷ Activate selected piezo
    if not self.in_collision(pos) then                    ▷ Check if clear of obstacles
        self.set_piezo_off()                              ▷ Deactivate piezo
        Break                                             ▷ Exit loop
```

Algorithm 3: Frame Skipping Implementation

```
Initialize obs_buffer, total_reward, done
for i in range (skip) do
    obs, reward, done, info ← env.step(action)            ▷Take action
    Store obs[image] in obs_buffer                        ▷Store for max pooling
    Total_reward += reward                                ▷Sum reward
    if done then
        Break                                             ▷Exit if done
Max_frame ← max(obs_buffer)                               ▷Max pooling
return max_frame, total_reward, done, info                ▷Return results
```

Legends for movies S1 to S7

Movie S1. Training process of MBRL in a real microfluidic racetrack channel. The left video shows early training (<100k steps) where the algorithm struggles to reach the target. On the right, after 340k steps, the algorithm shows improved target acquisition. The full process spans 10 days due to real-environment interactions.

Movie S2. Transfer learning behavior from a simulation environment to a real experimental environment of the same shape. It shows that the model converges in real experiments in just 3 hours.

Movie S3. Continuous action training in a vascular channel. The left side shows RRT* blue tree branches searching for the shortest path, marked in red when found. On the right, the microrobot is marked in blue, with the next target in red. The video demonstrates microrobots attempting to follow the path in real time.

Movie S4. Transfer learning from a simulation environment to a real vascular channel using an MBRL model with sweeping actions.

Movie S5. MBRL general model trained on 10 environments demonstrates its ability to perform across all 10 environments and adapt to a new, unseen channel with just 30 minutes of additional training.

Movie S6. Autonomous manipulation in a flow environment after transfer learning from a simulation that mimics the flow, guiding the microrobot to move in a low-drag region near the wall.

Movie S7. Active and passive shape-shifting of a microrobot navigating obstacles in a microchannel is demonstrated. Passive deformation occurs when a single piezoelectric transducer (PZT) is activated, while active manipulation involves dynamic shape changes using multiple PZTs for precise control and navigation.

Legends Extended Figures 1 to 4

Extended Figure 1 | Training with Continuous Actions. a. A plot displaying the relationship between the reward and the steps , illustrating the training progress and performance trends. The solid line represents the exponentially weighted moving average (EWMA) of the reward ($\alpha = 0.01$), and the shaded region shows ±½ of the rolling standard deviation (window = 50). **b.** RRT* path planning within an artificial vascular channel. **c.** A sequence of images showing the microrobot (blue) following the preplanned path (yellow) to reach updated targets (red). The updated targets and intermediate points are marked along the path to visualize the microrobot's tracking accuracy and performance. Scale bar: 300 µm.

Extended Figure 2 | MBRL Model Training and Adaptation. a. Simulated Randomized Environment Complexity: Displays six images, each at a different time step, showing the increasing complexity of simulated randomized environments with obstacles (black) and paths (white). The microrobot (red) navigates towards the target (blue) through these evolving challenges. **b**. Generalization Across Environments: Depicts adaptation over 12 environments, including 10 original plus 2 newly introduced randomized environments, culminating in a 70% success rate in a novel testing environment, highlighting effective generalization. Solid lines show the exponentially weighted moving average (EWMA, $\alpha = 0.01$) of success rates, with shaded areas indicating ±½ of the rolling standard deviation (window = 50 steps). A red dashed line marks the environment transition. The final box plot shows the post-adaptation performance, with boxes representing the 25th–75th percentile and whiskers extending to 1.5×IQR.

Extended Figure 3 | Microrobot Active and passive Shape-shifting , Demonstrated in Various Configurations: a. Schematic of the experimental setup, showing a straight microchannel equipped with four piezoelectric transducers (PZTs) positioned around an obstacle to manipulate the microrobot. **b.** Sequence of images illustrating passive microrobot deformation as it navigates through an obstacle using a single PZT activated along the X-axis at 20 volts, demonstrating the robot's ability to passively overcome the barrier. **c.** Series of images showing active manipulation where two PZTs on the Y-axis are activated, detailing the microrobot's dynamic shape deformation to navigate around the obstacle. **d.** Image sequence

in a bifurcation channel setup where the microrobot encounters an obstacle on one side and actively shape-shifts to maneuver towards the downward path, showcasing advanced control and navigational capabilities. Scale bar: 100 µm.

Extended Figure 4 | 3D Demonstration of Microrobot Manipulation: a. 3D microchannel where microrobots are manipulated. **b.** Bottom view of the transducer array, illustrating the arrangement of PZTs for 3D control. **c.** Experimental setup featuring an array of 18 piezoelectric transducers placed in a conical channel, with coupling gel applied between the array and the microchannel for efficient ultrasound transmission. **d.** Sequence of images showing microrobot movement in 3D as it leaves the focal plane, demonstrating its ability to navigate beyond the primary field of view.

References

1. Piscaglia, F. & Bolondi, L. The safety of Sonovue® in abdominal applications: Retrospective analysis of 23188 investigations. *Ultrasound in Medicine & Biology* **32**, 1369–1375 (2006).
2. Pelekasis, N. A., Gaki, A., Doinikov, A. & Tsamopoulos, J. A. Secondary Bjerknes forces between two bubbles and the phenomenon of acoustic streamers. *Journal of Fluid Mechanics* **500**, 313–347 (2004).
3. Doinikov, A. A. Acoustic radiation forces: Classical theory and recent advances. *Recent research developments in acoustics* **1**, 39–67 (2003).
4. Kirillov, A. *et al.* Segment Anything. Preprint at https://doi.org/10.48550/arXiv.2304.02643 (2023).
5. Hafner, D., Pasukonis, J., Ba, J. & Lillicrap, T. Mastering Diverse Domains through World Models. Preprint at https://doi.org/10.48550/arXiv.2301.04104 (2024).
6. Schulman, J., Wolski, F., Dhariwal, P., Radford, A. & Klimov, O. Proximal Policy Optimization Algorithms. Preprint at https://doi.org/10.48550/arXiv.1707.06347 (2017).
7. Mnih, V. *et al.* Human-level control through deep reinforcement learning. *Nature* **518**, 529–533 (2015).
8. Ha, D. & Schmidhuber, J. World Models. (2018) doi:10.5281/zenodo.1207631.
9. Thielicke, W. & Sonntag, R. Particle Image Velocimetry for MATLAB: Accuracy and enhanced algorithms in PIVlab. *Journal of Open Research Software* **9**, (2021).
